# Myelinating Schwann cells ensheath multiple axons in the absence of E3 ligase component Fbxw7

**DOI:** 10.1038/s41467-019-10881-y

**Published:** 2019-07-05

**Authors:** Breanne L. Harty, Fernanda Coelho, Sarah E. Pease-Raissi, Amit Mogha, Sarah D. Ackerman, Amy L. Herbert, Robert W. Gereau, Judith P. Golden, David A. Lyons, Jonah R. Chan, Kelly R. Monk

**Affiliations:** 1 Thaden School, 410 SE Staggerwing Lane, Bentonville, AR 72712 USA; 2 Department of Developmental Biology, Washington University School of Medicine, 660S. Euclid Ave., St. Louis, MO 63110 USA; 3 Vollum Institute, Oregon Health & Science University, 3181 SW Sam Jackson Park Rd., Portland, OR 97239 USA; 4 Department of Neurology, Weill Institute for Neuroscience, University of California San Francisco, 675 Nelson Rising Lane, San Francisco, CA 94158 USA; 5 Department of Anesthesiology, Washington University Pain Center, 660S. Euclid Ave., St. Louis, MO 63110 USA; 6 Centre for Brain Discovery Sciences, MS Society Centre for Translational Research, Euan MacDonald Centre for Motor Neurone Disease Research, University of Edinburgh, 49 Little France Crescent, Edinburgh, EH16 4SB UK; 7 Present Address: Institute of Neuroscience, University of Oregon, 1440 Franklin Blvd., Eugene, OR 97403 USA; 8 Present Address: Department of Developmental Biology, Stanford University, 279W. Campus Dr., Stanford, CA 94305 USA

**Keywords:** Cellular neuroscience, Schwann cell, Myelin biology and repair

## Abstract

In the central nervous system (CNS), oligodendrocytes myelinate multiple axons; in the peripheral nervous system (PNS), Schwann cells (SCs) myelinate a single axon. Why are the myelinating potentials of these glia so fundamentally different? Here, we find that loss of *Fbxw7*, an E3 ubiquitin ligase component, enhances the myelinating potential of SCs. *Fbxw7* mutant SCs make thicker myelin sheaths and sometimes appear to myelinate multiple axons in a fashion reminiscent of oligodendrocytes. Several *Fbxw7* mutant phenotypes are due to dysregulation of mTOR; however, the remarkable ability of mutant SCs to ensheathe multiple axons is independent of mTOR signaling. This indicates distinct roles for Fbxw7 in SC biology including modes of axon interactions previously thought to fundamentally distinguish myelinating SCs from oligodendrocytes. Our data reveal unexpected plasticity in the myelinating potential of SCs, which may have important implications for our understanding of both PNS and CNS myelination and myelin repair.

## Abstract (web summary)

The authors find that deletion from Schwann cells of an E3 ubiquitin ligase component called Fbxw7 leads to a phenotype reminiscent of myelination in the central nervous system where a single oligodendrocyte ensheaths multiple axons.

## Introduction

In vertebrates, oligodendrocytes (OLs) and Schwann cells (SCs) are specialized glial cells that generate the myelin sheaths of the central nervous system (CNS) and peripheral nervous system (PNS), respectively. In both the CNS and PNS myelin enables fast action potential propagation and protects the axon that it surrounds^1^. Myelin sheaths of the CNS and PNS are broadly similar in composition and structure, and there is a large degree of overlap in the molecular control of myelination by OLs and myelinating SCs^2,3^. However, there are important differences in how OLs and SCs interact with axons. One major fundamental difference between OLs and myelinating SCs is the ratio by which they myelinate axons. In peripheral nerves, SCs select specific axons in a process called radial sorting, whereby they are thought to extend exploratory processes into a bundle of unmyelinated axons and select just one axon, >1 μm in diameter, for myelination. Upon completion of radial sorting, promyelinating SCs are associated 1:1 with large-caliber axons (>1 μm in diameter), whereupon they initiate formation of a myelin sheath. In contrast, multiple small-caliber axons in peripheral nerves are ensheathed by non-myelinating Remak SCs into Remak bundles. The mechanisms controlling the Remak SC vs. myelinating SC fate are unclear. In the CNS, OLs also extend exploratory processes that dynamically interact with potential target axons^4^, but unlike SCs, OLs are able to myelinate many axon segments^5^. Given the broad molecular similarities between myelinating SCs and OLs, it is unclear why SCs and OLs exhibit such differences in myelinating capacity.

The E3 ubiquitin ligase component F-box and WD-repeat domain containing 7 (Fbxw7) is an important regulator of OL development and CNS myelination^6–8^. Here, using SC-specific knockout approaches in mice, we demonstrate that Fbxw7 plays distinct roles in SCs. Of principal interest is that, in the absence of Fbxw7, SCs appear to gain the ability to myelinate multiple axons in a fashion reminiscent of OLs in vivo and in vitro. Also surprising was the apparent ability of *Fbxw7* mutant SCs to generate myelin around large-caliber axons while simultaneously ensheathing many additional small-caliber axons. Electron microscopy and immunofluorescence analyses confirm that these cells are indeed SCs and not OLs that may have infiltrated the PNS. In addition, *Fbxw7* mutants display early increases in SC number, smaller Remak bundles, and hypermyelination which are ameliorated upon loss of *mTOR*. However, even in the absence of mTOR, *Fbxw7* mutant SCs appear to retain the ability to myelinate multiple axons, as well as simultaneously myelinate large axons while ensheathing small unmyelinated axons. This suggests that the molecular mechanisms that regulate the fundamental difference in myelinating potential between SCs and OLs are independent of mTOR signaling. Taken together, our findings show that the restriction of myelinating SCs to myelinate a single axon may be mutable and that Fbxw7 is a critical player in regulating the myelinating potential of SCs.

## Results

### Fbxw7 cell-autonomously regulates SC development

Fbxw7 is a substrate recognition component of SKP1-Cullin-F-box (SCF) ubiquitin ligase complexes, which catalyze addition of ubiquitin moieties on certain proteins to target them for proteasomal degradation^9^. We and others have previously reported that *fbxw7* zebrafish mutants display striking overexpression of *myelin basic protein* (*mbp*) in the CNS, enhanced OL numbers and thicker CNS myelin^6–8^.

Given the role of Fbxw7 in OLs, and the overlap in developmental programs that regulate OL versus SC maturation, we hypothesized that Fbxw7 is also required in SCs. To test this, we employed a conditional knockout strategy in mice. The *Dhh*^*cre*^ transgene results in Cre recombinase expression under the *Desert hedgehog* promoter at approximately embryonic day (E) 12.5 in SC precursors^10^. To delete *Fbxw7* specifically in SCs, we crossed *Dhh*^*cre*^ with an *Fbxw7*^*fl*/*fl*^ transgenic line in which *loxP* sites flank exons 5 and 6 of *Fbxw7*^11^. This creates a frameshift upon Cre activity resulting in a null allele, which we confirmed using RT-PCR to visualize the absence of mRNA (Supplementary Fig. 1a). *Dhh*^*cre*(+)^*;Fbxw7*^*fl*/*fl*^ conditional mutants were viable, fertile, and appeared grossly normal compared with controls. In all cases, *Dhh*^*cre*(*−*)^*;Fbxw7*^*fl*/+^ and *Dhh*^*cre*(*−*)^*;Fbxw7*^*fl*/*fl*^ siblings were used as controls.

**Fig. 1:**
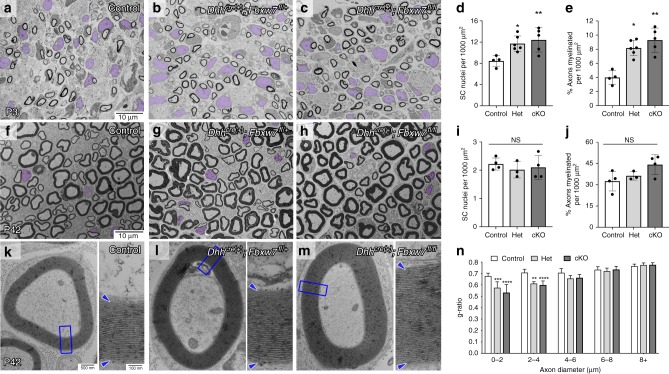
Fbxw7 is a cell autonomous regulator of SC number and myelin thickness. **a–j** Transmission electron micrographs (TEMs) and quantification of SC-specific *Fbxw7* mutant mice at P3 (**a–e**) and P42 (**f–j**). At P3, loss of *Fbxw7* leads to increased SC number (**d**; nuclei pseudocolored in purple in micrographs; *p* = 0.0224(*), *p* = 0.0015(**); one-way ANOVA) and percentage of myelinated axons (**e**; *p* = 0.0421(*), *p* = 0.0091(**), one-way ANOVA) in both *Dhh*^*cre*(+)^*;Fbxw7*^*fl*/+^ (Het [heterozygous]) and *Dhh*^*cre*(+)^*;Fbxw7*^*fl*/*fl*^ (cKO [conditional knockout]) nerves compared with littermate controls. However, by P42, both of these phenotypes have been resolved (**i**—Control to Het *p* = 0.7085, Control to cKO *p* = 0.8578, Het to cKO *p* = 0.9470; **j**—Control to Het *p* = 0.9583, Control to cKO *p* = 0.8507, Het to cKO *p* = 0.9719; one-way ANOVA for **i** and **j**). Mutant SCs also make thicker myelin as evidenced by decreased g-ratios (**k**–**n**), especially on small diameter axons (**n**; 0–2 μm—*p* = 0.0010(***), *p* < 0.0001(****); 2–4 μm—*p* = 0.0022(**), *p* < 0.0001(****); two-way ANOVA). Insets are zoomed images of the area indicated by blue boxes. Asterisks above bars indicate comparisons with controls. Comparisons between *Dhh*^*cre*(+)^*;Fbxw7*^*fl*/+^ and *Dhh*^*cre*(+)^*;Fbxw7*^*fl*/*fl*^ were not significant (*p* > 0.05). P3: *N* = 4 control, 6 *Dhh*^*cre*(+)^*;Fbxw7*^*fl*/+^, 5 *Dhh*^*cre*(+)^*;Fbxw7*^*fl*/*fl*^. P42: *N* = 4 control, 3 *Dhh*^*cre*(+)^*;Fbxw7*^*fl*/+^, 4 *Dhh*^*cre*(+)^*;Fbxw7*^*fl*/*fl*^. Error bars depict S.D.

We analyzed sciatic nerves by transmission electron microscopy (TEM) and found that at postnatal day 3 (P3), heterozygous *Dhh*^*cre*(+)^*;Fbxw7*^*fl***/+**^ (Het) and homozygous *Dhh*^*cre*(+)^*;Fbxw7*^*fl*/*fl*^ (cKO) mutant mice displayed an increase in SC nuclei relative to controls (Fig. 1a–d). However, by P42, SC numbers were equivalent in mutant and wild-type nerves (Fig. 1f–i), demonstrating that the early increase in SC number in *Fbxw7* mutants is transient. *Dhh*^*cre*(+)^*;Fbxw7*^*fl*/+^ and *Dhh*^*cre*(+)^*;Fbxw7*^*fl*/*fl*^ nerves also had a higher proportion of myelinated axons relative to control nerves (Fig. 1e, j), although there were no significant changes in total axon numbers, (Supplementary Fig. 1b).

The myelin was also considerably thicker in *Dhh*^*cre*(+)^*;Fbxw7*^*fl*/+^ and *Dhh*^*cre*(+)^*;Fbxw7*^*fl*/*fl*^ mice compared with controls beginning at P21, especially on small-caliber axons (Fig. 1k–n, Supplementary Fig. 1c, d). Importantly, however, mutant SCs do not appear to be myelinating axons <1 μm in diameter any more frequently than control SCs. This suggests that Fbxw7 mutant SCs are able to respond appropriately to Neuregulin signaling^12,13^. Finally, we found that Remak bundles contained fewer axons in *Dhh*^*cre*(+)^*;Fbxw7*^*fl*/+^ and *Dhh*^*cre*(+)^*;Fbxw7*^*fl*/*fl*^ nerves relative to controls (Supplementary Fig. 1e–s). These data demonstrate that Fbxw7 functions cell-autonomously to regulate multiple aspects of SC development.

### Fbxw7 mutant Schwann cells appear to myelinate multiple axons

**Fig. 2:**
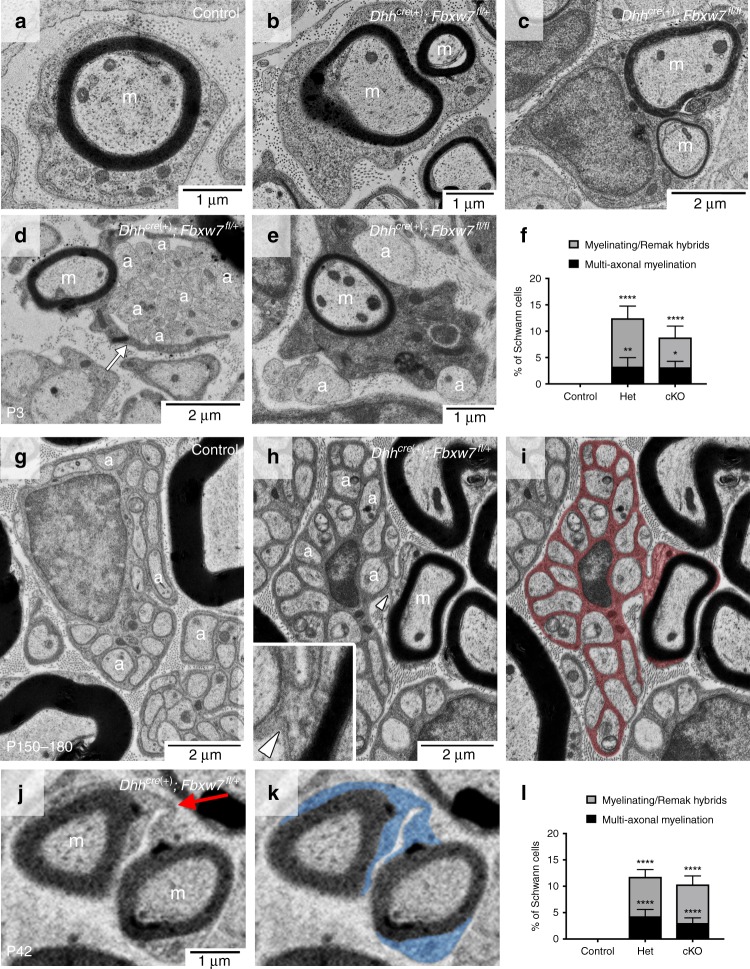
Loss of Fbxw7 enhances the myelinating capacity of SCs. TEM of SC-specific *Fbxw7* mutant mice show aberrant SC-axon interactions as early as P3 (**a**–**f**) and continuing until at least P180 (**g**–**l**). SCs lacking Fbxw7 appear to myelinate multiple axons (**b**, **c**, **j**) as well as generate myelin while simultaneously extending processes to encompass small-caliber non-myelinated axons (**d**, **e**, **h**, **i**). In Het nerves, ~4% of SCs myelinated multiple axons while another 7–8% appeared to be myelinating/Remak hybrids (**f**, **l**; Het multi-axonal myelination *p* = 0.0063 (P3) *p* < 0.0001 (P150–180); Het myelinating/Remak hybrids *p* < 0.0001 (P3 and P150–180)). The percentage of SCs displaying these phenotypes in cKO nerves was similar at both P3 and P150–180 (**f**, **i**; cKO multi-axonal myelination *p* = 0.0382 (P3), *p* < 0.0001 (P150–180); cKO myelinating/Remak hybrids *p* < 0.0001 (P3 and P150–180); ANOVA). In both phenotypes there was continuous basal lamina (white arrowhead) around these cells and their processes (**h**, inset), and it was the outer cytoplasmic pocket that extended these additional processes (**d**; white arrow). In aged nerves, some SCs still appeared to be myelinating/Remak hybrids (**i**; SC cytoplasm pseudocolored in red). The true proportion of mutant SCs displaying these behaviors is likely underestimated by cross-sectional analyses (**f**, **l**) as multiple myelinated axons appeared to be joined by very thin cytoplasmic processes (**k**; SC cytoplasm pseudocolored in blue). P3: *N* = 4 control, 6 *Dhh*^*cre*(+)^*;Fbxw7*^*fl*/+^, 5 *Dhh*^*cre*(+)^*;Fbxw7*^*fl*/*fl*^. P150–180: *N* = 5 control, 4 *Dhh*^*cre*(+)^*;Fbxw7*^*fl*/+^, 5 *Dhh*^*cre*(+)^*;Fbxw7*^*fl*/*fl*^. Asterisks above bars indicate comparisons with controls; comparisons between *Dhh*^*cre*(+)^*;Fbxw7*^*fl*/+^ and *Dhh*^*cre*(+)^*;Fbxw7*^*fl*/*fl*^ were not significant (*p* > 0.05). m = myelinated axon; a = unmyelinated axon. Error bars depict S.D.

We also found that loss of Fbxw7 dramatically increased the myelinating potential of SCs (Fig. 2, Supplementary Figs. 2 and 3). In every *Dhh*^*cre*(+)^*;Fbxw7*^*fl*/+^ and *Dhh*^*cre*(+)^*;Fbxw7*^*fl*/*fl*^ nerve examined, even in adulthood, we observed numerous instances of what appeared to be multi-axonal myelination (Fig. 2b, c, f, j, k; Supplementary Fig. 2a–e). These myelinated axons were sometimes joined by long thin processes of SC cytoplasm in a manner reminiscent of OL myelination (Fig. 2j, red arrow).

**Fig. 3:**
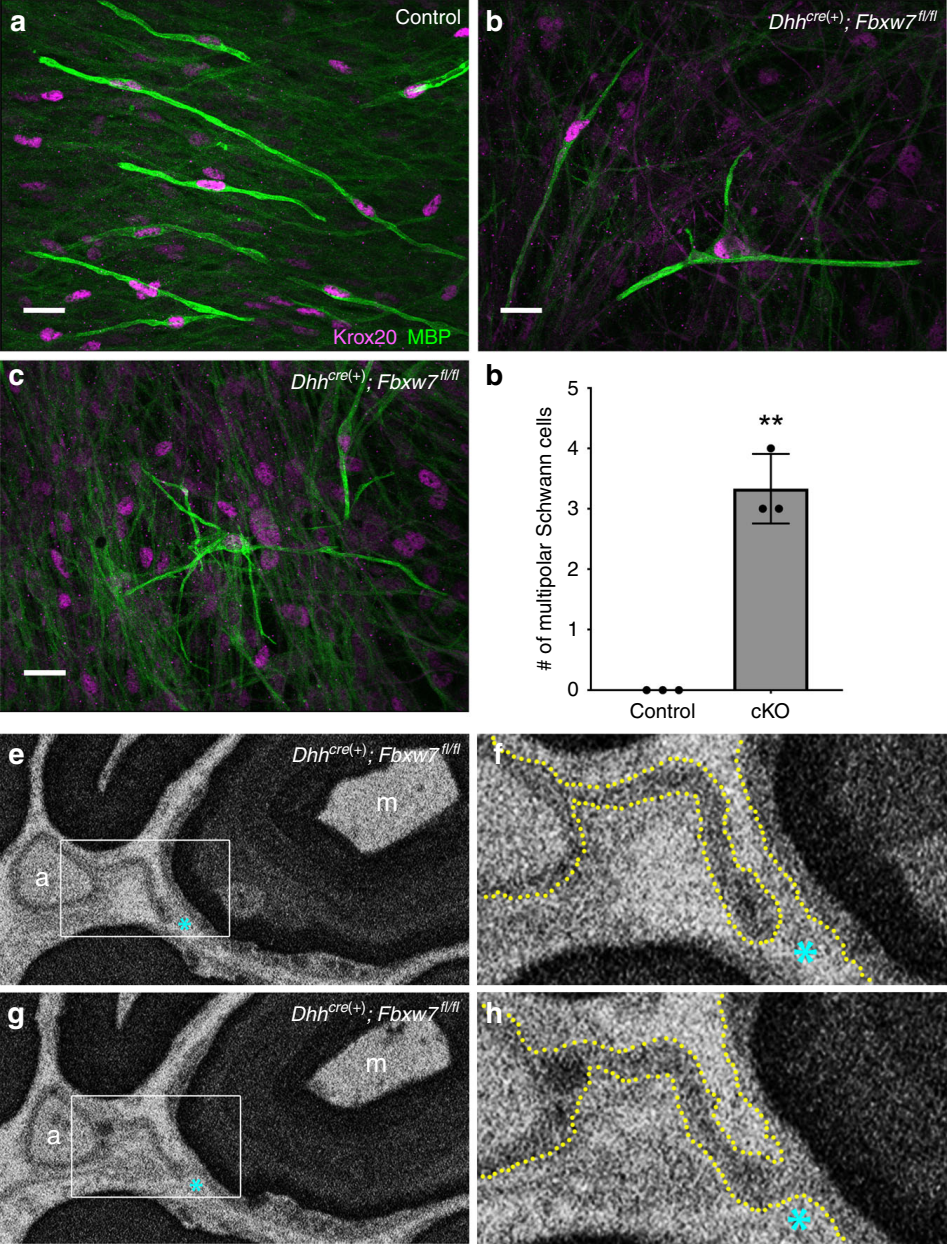
*Fbxw7* mutant SCs display branched 3D morphology. Dissociated SC/DRG co-cultures from control (**a**) and *Dhh*^*cre*(+)^*:Fbxw7*^*fl*/*fl*^ (**b**, **c**) mouse embryos indicate that WT MBP(+) SCs are bipolar (**a**), whereas mutant SCs can assume a multipolar morphology (**b**) sometimes with highly branched processes (**c**). These phenotypes did not occur in the control cultures (**d**; *p* = 0.0099, unpaired *t*-test with Welch’s correction). Data are quantified per 490.88 mm^2^ and represent two technical replicate cultures from each of three independent mouse embryos per genotype. Scale bar is 20 μm. Serial block-face scanning electron microscopy (SBF-SEM) images of a *Dhh*^*cre*(+)^*:Fbxw7*^*fl*/*fl*^ nerve at P150 (**e**–**h**). A mutant SC extends a thin cytoplasmic process between an unmyelinated axon (marked by a) to a myelinated axon (marked by m) (**e**; white box denotes the area shown in (**f**); cyan asterisk is for reference). In some sections we see that the process is distinct and does not touch the membrane around the myelinated axon (**f**; yellow dotted line denotes cell membranes; cyan asterisk for reference). However, in other sections it is clear that these two axons are joined by continuous cytoplasm (**g**, **h**). SBF-SEM images shown here are stills from Supplementary Movie 1. Error bars depict S.D.

We also observed *Fbxw7* mutant SCs that had myelinated one or more axons and appeared to simultaneously encompassed several unmyelinated axons in an immature SC or Remak SC-like fashion (Fig. 2d–f; Supplementary Fig. 2f–j). When mature, these SCs displayed both myelinating SC and Remak SC qualities almost as though these cells are myelinating/Remak SC hybrids, with the myelin being grossly normal and every unmyelinated axon being fully ensheathed by SC membrane and cytoplasm (Fig. 2h, Supplementary Fig. 2h–j). Importantly, both of these distinct phenotypes were observed at all developmental stages examined—P3, P21, P42, and P150–180—suggesting that they are not simply transient developmental anomalies. It is also important to highlight that these phenotypes are distinct from polyaxonal myelination, in which a bundle of axons is myelinated together as though it was one larger axon (Supplementary Fig. 2k), which has been previously reported in mutants with radial sorting defects and occurs at low frequency even in wild-type nerves^14^.

In total, by cross-sectional TEM analyses, ~12% of *Fbxw7* mutant SCs display enhanced myelinating potential, and this proportion remained consistent throughout the life of the animal (Fig. 2f, l). In many cases, it appeared that multiple myelinated axons (Fig. 2j; red arrow) or a myelinated axon and a bundle of unmyelinated axons (Supplementary Fig. 2g,j; blue arrow), were joined together by long thin processes of SC cytoplasm. This suggests that mutant SCs may be assuming a branched 3D morphology and that static cross-sectional imaging may not give a complete representation of the phenotype. Furthermore, 2D imaging may underestimate the proportion of SCs exhibiting enhanced myelinating potential.

To visualize the 3D architecture of mutant SCs, we took two parallel approaches. To examine the myelinating potential of individual SCs lacking *Fbxw7*, we prepared dissociated SC/dorsal root ganglia sensory neuron co-cultures from *Dhh*^*cre*(+)^*;Fbxw7*^*fl*/*fl*^ and littermate control mouse embryos. In all *Dhh*^*cre*(+)^*;Fbxw7*^*fl*/*fl*^ co-cultures we observed a low incidence of MBP+/Krox20 + SCs with more than three MBP+ processes, which did not appear in any of the control conditions (Fig. 3a–d). In a few instances *Fbxw7* mutant SCs displayed a highly branched morphology and appeared to interact with more than five axons simultaneously (Fig. 3c).

We also performed serial block-face scanning electron microscopy (SBF-SEM) to visualize a mutant nerve in 3D (N = 1 *Dhh*^*cre*(+)^*;Fbxw7*^*fl*/*fl*^ nerve; 11 different regions within the nerve). We found that in most individual cross-sections, myelinating cells appear to display the normal 1:1 SC:axon relationship. However, when we assessed the 3D architecture of different portions of the mutant nerves, it became clear that *Fbxw7* mutant SCs that look normal in one plane display phenotypes indicative of enhanced myelinating potential in other sections. For example, an *Fbxw7* mutant non-myelinating SC is observed extending a thin cytoplasmic process (Fig. 3e) in one plane that appears to join a myelinated axon in another plane (Fig. 3f; Supplementary Movie 1).

### Multipolar cells in *Fbxw7* mutant nerves are not OLs

In order to test the possibility that the multipolar SCs observed in *Fbxw7* mutants might represent OLs that migrated into peripheral nerves, we asked whether the aberrantly myelinating cells in our mutant nerves had characteristics of bona fide SCs or OLs. Importantly, in both types of aberrant SC-axon interactions in *Fbxw7* mutants, we were able to trace continuous SC cytoplasm and basal lamina on the abaxonal surface of the mutant SC (e.g., Fig. 2h, inset, white arrowhead). SCs secrete a basal lamina and OLs do not; thus, the presence of a basal lamina strongly supports the notion that these cells are indeed SCs and not OLs that might have infiltrated the PNS. In addition, our co-culture experiments clearly demonstrate the ability of MBP+/Krox20+ cells (a molecular signature of SCs) to myelinate multiple axonal segments (Fig. 3a–c). These data suggest that the enhanced myelinating potential observed in *Fbxw7* mutants is not due to OL infiltration. Together with the morphological analyses of *Fbxw7* mutant SCs in vivo and in vitro, these data suggest that SCs lacking Fbxw7 become multipolar and myelinate or interact with multiple axons simultaneously in a manner reminiscent of OL myelination.

### Loss of *Fbxw7* in SCs causes mild motor and sensory deficits

Given the ultrastructural phenotypes observed in *Dhh*^*cre*(+)^*;Fbxw7*^*fl*/+^ and *Dhh*^*cre*(+)^*;Fbxw7*^*fl*/*fl*^ nerves, we next assessed the functional consequences of loss of Fbxw7 in SCs. To this end, we performed a battery of motor and sensory behavioral analyses using *Dhh*^*cre*(+)^*;Fbxw7*^*fl*/*fl*^ mice and their littermate controls at 6 months of age (Supplementary Fig. 3a–k). We found that the *Dhh*^*cre*(+)^*;Fbxw7*^*fl*/*fl*^ mice displayed modest behavioral deficits. In the vertical pole test, *Dhh*^*cre*(+)^*;Fbxw7*^*fl*/*fl*^ mice took longer to climb down the pole than their control siblings, suggesting a mild defect in complex motor behavior^15,16^ (Supplementary Fig. 3e, f). Through gait analyses, we also found that there was a very small but significant decrease in the maximum contact intensity (Supplementary Fig. 3g) and print area (Supplementary Fig. 3h) of the fore paws of *Dhh*^*cre*(+)^*;Fbxw7*^*fl*/*fl*^ mice. In addition, mutant animals trended towards a mild hypersensitivity to cold induced by the evaporative cooling of acetone applied to the hind paw^17^ (Supplementary Fig. 3k), although this result did not reach statistical significance (*p* = 0.0977).

### Fbxw7 regulates mTOR to control SC number, myelination, and Remak bundle organization

To our knowledge, no genetic or pharmacological manipulation in vivo has been reported to increase the myelinating ensheathment potential of SCs in the manner observed in *Fbxw7* mutants. However, the hypermyelination and defects in Remak bundle size observed in *Fbxw7* mutants have been described following SC-specific deletion of *Pten*^18^ or overactivation of Akt^19^, which both result in elevated mTOR signaling. Previous reports show loss of Fbxw7 function enhances levels of mTOR and its targets^20^, and Fbxw7 was recently shown to regulate mTOR in CNS myelination^7^.

**Fig. 4:**
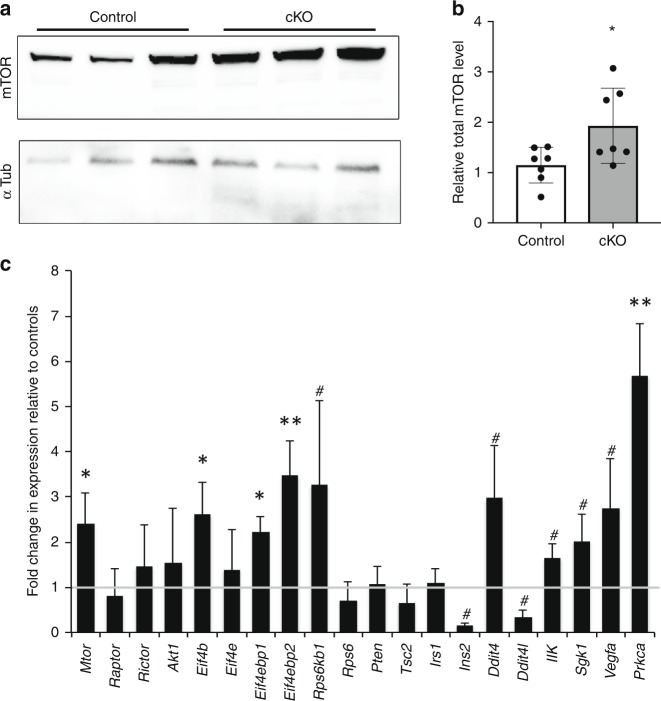
mTOR levels are elevated in *Fbxw7* mutants. Western blot analyses of sciatic nerve lysates indicate a 2-fold increase in mTOR protein in *Dhh*^*cre*(+)^*:Fbxw7*^*fl*/*fl*^ (cKO [conditional knockout]) nerves compared with littermate controls when normalized to both background and alpha-tubulin levels (**a**, **b**; *p* = 0.0350, unpaired *t*-test with Welch’s correction). *mTOR* mRNA is also upregulated in *Dhh*^*cre*(+)^*:Fbxw7*^*fl*/*fl*^ nerves (**c**; mTOR *p* = 0.0236, Eif4b *p* = 0.417, Eif4ebp1 *p* = 0.0398, Eif4ebp2 *p* = 0.0087, Prkca *p* = 0.0073; # = *p* < 0.1; one-way ANOVA), as are multiple mTOR targets. The gray line at *y* = 1 denotes control levels. For western blots: *N* = 5 controls, *N* = 5 *Dhh*^*cre*(+)^*:Fbxw7*^*fl*/*fl*^ at P42. For qPCR, *N* = 3 control and 3 *Dhh*^*cre*(+)^*:Fbxw7*^*fl*/*fl*^ at P21. Error bars depict S.D.

Therefore, we examined levels of mTOR and found that total mTOR protein levels are ~2-fold higher in *Fbxw7* mutants relative to controls (Fig. 4a, b). Similarly, and consistent with previous findings that multiple feedback loops regulate the PI3K/mTOR pathway^21^, we found that the mRNA levels of mTOR as well as several targets of mTOR were also significantly elevated in *Fbxw7* mutant nerves (Fig. 4c).

**Fig. 5:**
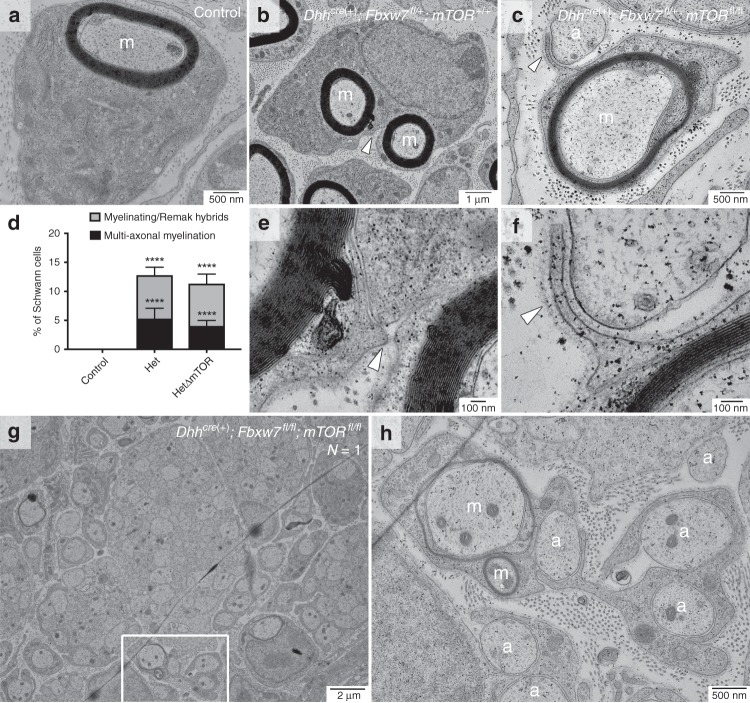
The ensheathment capacity of myelinating SCs is independent of mTOR signaling. *mTOR* deletion in *Fbxw7* mutant nerves failed to suppress aberrant SC-axon interactions. Apparent multi-axonal myelination and SCs that appeared to both myelinate and encompass multiple small non-myelinated axons were present in both *Dhh*^*cre*(+)^*;Fbxw7*^*fl*/+^*;mTOR*^+/+^ (**b**, **e**) and *Dhh*^*cre*(+)^*;Fbxw7*^*fl*/+^*;mTOR*^*fl*/*fl*^ (HetΔmTOR) (**c**, **f**) nerves. Neither phenotype was ever observed in sibling controls (**a**, **d**; *p* < 0.0001(****); ANOVA). Tracing the BL (**e**, **f**; white arrows) suggested these behaviors were the acts of single SCs. Both phenotypes were also present in the sole *Dhh*^*cre*(+)^*;Fbxw7*^*fl*/*fl*^*;mTOR*^*fl*/*fl*^ (cKOΔmTOR) animal we were able to collect (**g**, **h**). Asterisks above bars indicate comparisons with controls; comparisons between *Dhh*^*cre*(+)^*;Fbxw7*^*fl*/+^ and *Dhh*^*cre*(+)^*;Fbxw7*^*fl*/+^*;mTOR*^*fl*/*fl*^ were not significant. P3: *N* = 4 control, 6 *Dhh*^*cre*(+)^*;Fbxw7*^*fl*//+^*;mTOR*^+/+^, 5 *Dhh*^*cre*(+)^*;Fbxw7*^*fl*/+^*;mTOR*^*fl*/*fl*^, 1 *Dhh*^*cre*(+)^*;Fbxw7*^*fl*/*fl*^*;mTOR*^*fl*/*fl*^. m = myelinated axon; a = unmyelinated axon. Error bars depict S.D.

To directly test if Fbxw7 controls mTOR in SCs, we generated double SC-specific *Fbxw7;mTOR* knockouts by crossing *mTOR*^*fl*/*fl*^ mice^22^ to our *Dhh*^*cre*(+)^*;Fbxw7*^*fl*/*fl*^ mice (Supplementary Figs. 4 and 5). We were unable to obtain enough *Dhh*^*cre*(+)^*;Fbxw7*^*fl*/*fl*^*;mTOR*^*fl*/*fl*^ mutants from trans-heterozygous intercrosses (1/32 expected, 1/126 obtained (Fig. 5g, h)); therefore, we analyzed *Dhh*^*cre*(+)^*;Fbxw7*^*fl*/+^*;mTOR*^*fl*/*fl*^ (HetΔmTOR) animals in more detail as the *Fbxw7* mutation is dominant. For the epistasis experiments, *Dhh*^*cre*(−)^*;Fbxw7*^*fl*/+^*;mTOR*^*fl*/+^ and *Dhh*^*cre*(−)^*;Fbxw7*^*fl*/+^*;mTOR*^*fl*/*fl*^ siblings were used as controls.

If mTOR is the Fbxw7 target responsible for the SC defects observed in *Fbxw7* mutants, loss of mTOR should suppress these phenotypes in *Dhh*^*cre*(+)^*;Fbxw7*^*fl*/+^ animals. Indeed, at P3, *Dhh*^*cre*(+)^*;Fbxw7*^*fl*/+^*;mTOR*^*fl*/*fl*^ animals were indistinguishable from controls with regard to SC numbers and the percentage of myelinated axons (Supplementary Fig. 4a–e). At P21, Remak bundles in *Dhh*^*cre*(+)^*;Fbxw7*^*fl*/+^*;mTOR*^*fl*/*fl*^ nerves contained significantly more axons relative to Remak bundles in either controls or *Fbxw7* mutants (Supplementary Fig. 4f–j). Further, in agreement with previous studies^23^, *Dhh*^*cre*(+)^*;Fbxw7*^*fl*/+^*;mTOR*^*fl*/*fl*^ nerves were hypomyelinated relative to controls and *Dhh*^*cre*(+)^*;Fbxw7*^*fl*/+^ animals (Supplementary Fig. 4k), demonstrating that *mTOR* is epistatic to *Fbxw7*. These data suggest that Fbxw7 regulates mTOR to control early SC number, axon ensheathment by Remak SCs, and myelin thickness.

### SC myelinating potential is independent of mTOR signaling

Given that mTOR mediated the many of the SC developmental defects observed in *Fbxw7* mutants we next tested whether it also mediated the phenotype of multiple axon ensheathment by myelinating SCs. Strikingly, loss of *mTOR* in *Fbxw7* mutant SCs appeared unable to restore normal SC myelinating potential. Despite the fact that *Dhh*^*cre*(+)^*;Fbxw7*^*fl*/+^*;mTOR*^*fl*/*fl*^ nerves had normal numbers of SCs, delayed radial sorting, larger Remak bundles, and thinner myelin, loss of mTOR appeared to be insufficient to suppress the enhanced myelinating potential observed in *Fbxw7* mutant SCs. Double mutant myelinating SCs were still seen ensheathing multiple axons, as well as displaying myelinating/Remak hybrid phenotypes (Fig. 5). As in the *Dhh*^*cre*(+)^*;Fbxw7*^*fl*/+^ and *Dhh*^*cre*(+)^*;Fbxw7*^*fl*/*fl*^ animals, mutant SCs in *Dhh*^*cre*(+)^*;Fbxw7*^*fl*/+^*;mTOR*^*fl*/*fl*^ and *Dhh*^*cre*(+)^*;Fbxw7*^*fl*/*fl*^*;mTOR*^*fl*/*fl*^ nerves that had already myelinated large axons were seen extending processes from the outer cytoplasmic pocket to interact with other axons (Fig. 5c, f; white arrowhead). These data suggest that Fbxw7 controls SC myelinating potential independent of mTOR signaling. It is remarkable that SC myelinating capacity appears to remain enhanced in *Dhh*^*cre*(+)^*;Fbxw7*^*fl*/+^*;mTOR*^*fl*/*fl*^ mutants despite the fact that deletion of *mTOR* caused several defects in peripheral nerve development. This suggests that the morphological processes controlling the myelinating potential of SCs are distinct from the cellular behaviors involved in radial sorting, myelination, or Remak SC ensheathment.

### c-Jun is elevated in *Fbxw7* mutant SCs

**Fig. 6:**
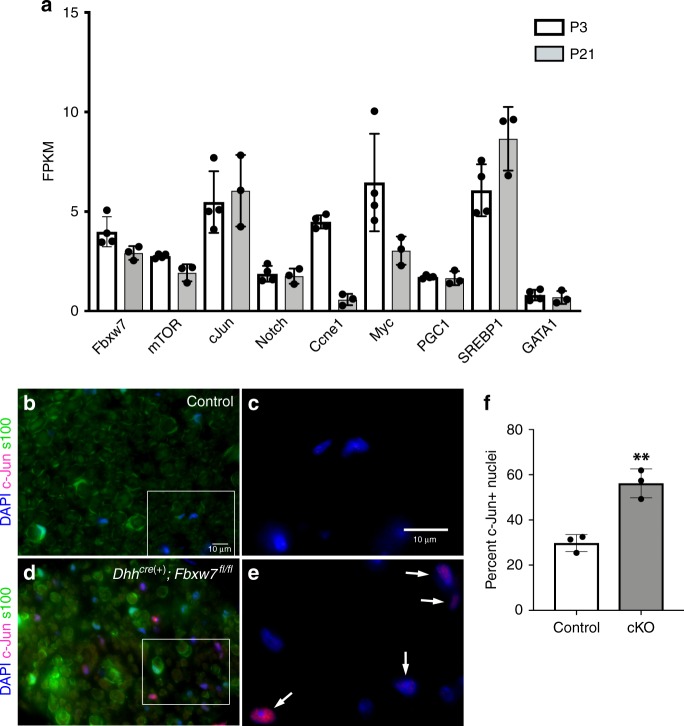
c-Jun is elevated in *Fbxw7* mutant nerves. Previously published RNAseq data^24^ from WT sciatic nerves at P3 and P21 was mined to reveal that, in addition to *Fbxw7* itself and *mTOR*, several other well-known targets of Fbxw7 are expressed during SC development (**a**). Immunofluorescence studies of control (**b**, **c**) and *Dhh*^*cre*(+)^*;Fbxw7*^*fl*/*fl*^ (**d**, **e**; white arrows indicate c-Jun positive nuclei; cKO) nerves at 6 months. cKO nerves display nearly three times the proportion of c-Jun + nuclei relative to controls (**f**; *p* = 0.0025 (**), unpaired *t*-test with Welch’s correction). *N* = 3 per genotype. Error bars depict S.D.

To begin to assess the possible molecular mechanism underlying the enhanced ensheathing potential of mutant myelinating SCs, we mined previously generated RNA sequencing (RNAseq) data from wild-type sciatic nerves at P3 and P21^24^ to determine if any other Fbxw7 targets are expressed in SCs. In addition to mTOR, we found that several other well-known targets of Fbxw7 including c-Jun^25^, Notch, and Cyclin E are also expressed in normal sciatic nerves at P3 and P21 (Fig. 6a). c-Jun stood out among these possible targets because it is required for the acquisition of the repair SC phenotype^26–28^, the multipolar morphology assumed by *Fbxw7* mutant SCs is reminiscent of branched repair SCs^29^, and loss of Fbxw7 has previously been shown to elevate c-Jun levels^30^. Therefore, we tested the hypothesis that c-Jun is elevated in *Dhh*^*cre*(+)^*;Fbxw7*^*fl*/*fl*^ relative to controls. Indeed, immunofluorescent analysis of c-Jun levels at P150–180 suggests that *Dhh*^*cre*(+)^*;Fbxw7*^*fl*/*fl*^ nerves display a greater proportion of c-Jun positive nuclei than sibling control nerves (Fig. 6b–f). Future epistasis experiments are required to test if inappropriate elevation of c-Jun causes enhanced myelinating potential in *Fbxw7* mutant SCs, and aberrant c-Jun levels may explain the morphological similarities between repair SCs and SCs that have lost Fbxw7 function.

**Fig. 7:**
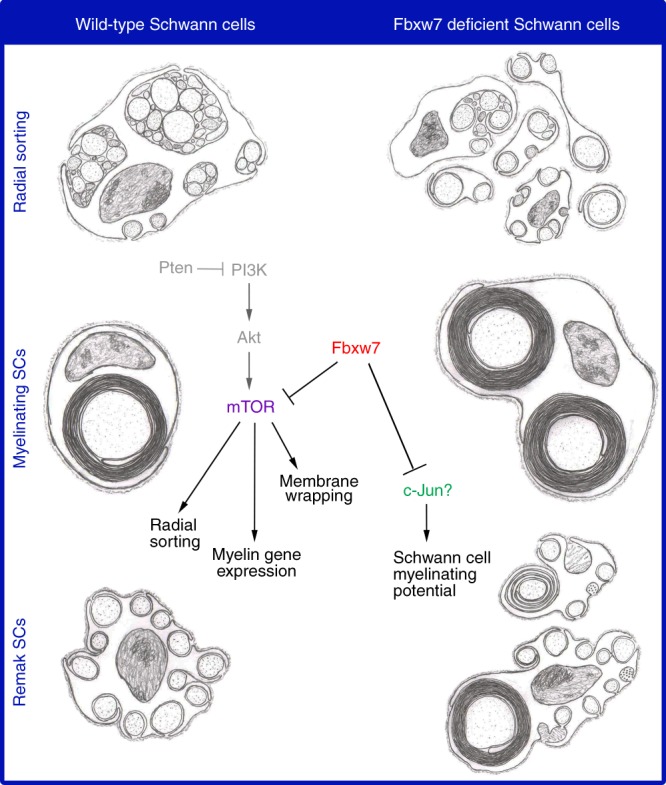
Fbxw7 orchestrates SC biology via mTOR-dependent and -independent mechanisms. Artistic renditions of the phenotypes observed in *Fbxw7* mutant SCs (right) as compared with normal SCs (left). Fbxw7 is involved in radial sorting (top), as well as both mature myelinating SCs (middle) and Remak SCs (bottom). A simplified PI3K/mTOR pathway shows that in SCs, Fbxw7 directly inhibits mTOR, and thus regulates multiple aspects of SC biology. Fbxw7 may regulate SC myelinating potential through its control of c-Jun. Steps in the pathway that were not demonstrated directly in this study are shown in gray. Pencil sketches by B.L.H.

Altogether, our data suggest that Fbxw7 inhibits SC myelinating potential such that loss of Fbxw7 function allows SCs to assume a multipolar branched morphology and to interact with and appear to myelinate axons in a fashion reminiscent of OL myelination. Further, we have shown here that this function of Fbxw7 is independent of its regulation of mTOR, which controls myelin thickness and some aspects of Remak SC biology. Given that *Fbxw7* mutant SCs are morphologically similar to repair SCs and that c-Jun, a well-known regulator of the repair SC phenotype, is elevated in mutant SCs, our current working model is that Fbxw7 may regulate myelinating SC ensheathment potential through inhibition of c-Jun (Fig. 7). However, Fbxw7 is known to control many different master regulators, many of which are also expressed in SCs at similar times (Fig. 6a). Although most have not yet been experimentally confirmed, ~1700 proteins contain one or more Fbxw7 degron motifs^31^ and thus are potential targets of Fbxw7. Therefore, significant additional research will be required to fully define the mechanisms underlying the regulation of SC myelinating potential by Fbxw7.

## Discussion

In stark contrast to OLs in the CNS, which can myelinate dozens of axon segments simultaneously, myelinating SCs are restricted to myelinating a single axonal segment in the PNS. The molecular mechanisms controlling the differences in myelinating potential between SCs and OLs remain mysterious. Here we show that SCs are capable of myelinating multiple axons in vivo and challenge the notion that the ability of a SC to extend multiple processes is mutually exclusive with the capacity to make myelin. Remak SCs are also capable of extending multiple processes and interacting with many axons, but unlike OLs they do not myelinate axons. However, upon loss of *Fbxw7*, single SCs can simultaneously myelinate some axons, while also appearing to encompass unmyelinated axons as if mutant SCs are hybrids between myelinating SCs and Remak SCs. Further, the multipolar, branched 3D morphology and apparent multi-axonal myelination observed in *Fbxw7* mutant SCs is reminiscent of OL myelination. However, the presence of basal laminae and presence of SC-lineage markers suggests that OLs have not infiltrated *Fbxw7* mutant nerves.

Although neither of the aberrant *Fbxw7* mutant SC-axon interaction phenotypes have been previously described in vivo, several other phenotypes observed in *Fbxw7* mutant nerves resemble phenotypes described in mutants where mTOR signaling is enhanced such as in *Pten* mutants^18^ and constitutively active *Akt* mutants^19^. It is well documented, from these studies and others, that mTOR levels must be tightly regulated in SCs such that any type of manipulation results in defective PNS myelination^18,19,23,32–37^. mTOR is a bona fide target of Fbxw7 in other contexts^20^, and Fbxw7 was recently shown to control OL myelination through mTOR^7^. Thus, we tested the hypothesis that Fbxw7 regulates mTOR to control SC development. We showed that mTOR and some of its targets are upregulated in *Fbxw7* mutants, suggesting that mTOR activity is elevated. Double transgenic analyses demonstrated that *mTOR* is epistatic to *Fbxw7* and is responsible for regulating early SC numbers, Remak bundle organization, and appropriate myelin thickness.

Notably, however, loss of *mTOR* in *Fbxw7* mutant SCs did not restore typical SC:axon ratios. Despite the fact that *Dhh*^*cre*(+)^*;Fbxw7*^*fl*/+^*;mTOR*^*fl*/*fl*^ nerves had normal numbers of SCs, delayed radial sorting, larger Remak bundles, and thinner myelin, loss of *Fbxw7* was nevertheless sufficient to appear to drive increased myelinating potential of SCs. This suggests that the myelinating potential of SCs is independent of mTOR signaling, and importantly, that the mechanisms controlling the typical SC:axon ratio is also controlled independently from other morphological behaviors of SCs, including radial sorting, Remak SC ensheathment, and membrane wrapping.

When we mined RNAseq data, we found that several well-known targets of Fbxw7 are also expressed in SCs during early development. One of these was the transcription factor c-Jun, which is an important molecular switch that must be upregulated after injury for SCs to properly assume repair SC phenotypes^27,28^. Interestingly, repair SCs adopt a branched morphology during nerve regeneration^29^ that resembles the multipolar 3D architecture we observed in *Fbxw7* mutant SCs. Although it is expressed at a low level in SCs, c-Jun is dispensable in normal nerves and thus c-Jun protein is often barely detectable except in cases of nerve injury^27,28^. Recent evidence also suggests that mTOR is transiently reactivated after nerve injury to promote the elevation of c-Jun^33^. Therefore, we hypothesized that c-Jun might be aberrantly upregulated upon loss of *Fbxw7*, possibly leading mutant SCs to assume a branched morphology similar to that of repair SCs. Indeed, we found that c-Jun levels in nerves from *Dhh*^*cre*(+)^*;Fbxw7*^*fl*/*fl*^ mice were nearly three times that of controls. Thus, future efforts will determine if developmental regulation of c-Jun by Fbxw7 controls SC myelinating potential. However, our work highlights the complicated nature of losing function of a protein like Fbxw7, which is a regulator of master regulators. Given the role of Fbxw7 in E3 ubiquitin ligase complexes, it is possible that dysregulation of c-Jun is causal for the aberrant SC phenotypes we observe. It is also possible that the complex phenotypes present in *Fbxw7* mutant nerves result from combinatorial interactions amongst multiple misregulated targets.

Future work should also explore the implications of enhanced myelinating potential on remyelination after PNS injury. In contrast to SC remyelination, OL remyelination and CNS recovery after injury is limited in mammals, and OLs do not de/redifferentiate to aid in recovery^38^. One interesting hypothesis is that the distinction between the ability of SCs vs. OLs to facilitate repair after injury is rooted in fundamental qualities that distinguish these cells, such as the number of axons they associate with and myelinate. It is now clear that OLs interpret and respond to neuronal activity by selective myelination and that OLs play a critical role in circuit development, refinement and/or maintenance^39–42^. It is intriguing to speculate that OLs may link circuits through their interactions with and myelination of multiple axons. However, in the PNS, where circuits are less complex, there is not strong evidence for SC participation in active modulation of circuit function. Rather, more emphasis seems to be placed on the ability of SCs to rapidly and faithfully respond to injuries, which occur more readily in the PNS. Presumably, this rapid response would be more difficult if SCs were required to demyelinate more than one axon, especially if only some of those axons were injured, while others were intact. *Fbxw7* mutants represent a unique and useful tool with which to investigate the impact of differences between myelinating SCs, Remak SCs, and OLs on nervous system repair. Moreover, our work demonstrates a previously unknown plasticity of SCs and suggests that the demarcation between the cell biology of SC and OL myelination may be less rigid than previously appreciated.

## Methods

### Contact for reagents and resource sharing

Requests for further information, resources, and reagents should be directed to and will be fulfilled by Kelly Monk (monk@ohsu.edu).

### Experimental model and subject details

All animal experiments were performed in compliance with institutional ethical regulations for animal testing and research at Washington University, Oregon Health and Science University (OHSU), and University of California San Francisco (UCSF). Experiments were approved by the Animal Care and Use Committee of Washington University School of Medicine (St. Louis, MO), the Institutional Animal Care and Use Committee of OHSU (Portland, OR), and the Institutional Animal Care and Use Program of UCSF (San Francisco, CA).

The Fbxw7 conditional-ready mice (*Fbxw7*^*fl*/*fl*^)^11^ were obtained from Jackson laboratories (Stock #: 017563) on a pure C57BL/6 background. *Fbxw7*^*fl*/*fl*^ mice were mated to *Dhh*^*Cre*(+)^ mice^10^ that had also been maintained on a pure C57BL/6 background (>7 generations) to generate *Dhh*^*Cre*(+)^*;Fbxw7*^*fl*/+^ (Het) mice. *Fbxw7* Hets were backcrossed to *Fbxw7*^*fl*/*fl*^ animals to obtain *Dhh*^*Cre*(+)^*;Fbxw7*^*fl*/*fl*^ (cKO) mice. For all cKO experiments, we used *Dhh*^*Cre*(−)^*;Fbxw7*^*fl*/+^ or *Dhh*^*Cre*(−)^*;Fbxw7*^*fl*/*fl*^ littermates as controls. For the double mutant experiments with Fbxw7 and mTOR, we obtained mTOR conditional-ready (*mTOR*^*fl*/*fl*^) mice^43^ from Jackson laboratories (Stock #: 0110009), also on a pure on C57BL/6 background. The *mTOR*^*fl*/*fl*^ mice were crossed with *Dhh*^*Cre*(+)^*;Fbxw7*^*fl*/*fl*^ animals to generate *Dhh*^*Cre*(+)^*;Fbxw7*^*fl*/+^*;mTOR*^*fl*/+^ animals. Finally, to obtain double mutants, we crossed *Dhh*^*Cre*(+)^*;Fbxw7*^*fl*/+^*;mTOR*^*fl*/+^ to *Dhh*^*Cre*(−)^*;Fbxw7*^*fl*/+^*;mTOR*^*fl*/+^ animals and analyzed *Dhh*^*Cre*(+)^*;Fbxw7*^*fl*/+^*;mTOR*^*fl*/*fl*^ animals (“HetΔmTOR” for brevity). In all cases, mice of both sexes were analyzed, in equal ratios whenever possible. In all cases mutants were compared with littermate sibling controls.

All mouse lines were genotyped as previously described^10,11,43^.

### Transmission electron microscopy (TEM)

TEM was performed on mouse sciatic nerves at P3, P21, P42 and ≥6 months^44^. Nerves were immersion-fixed in modified Karnovsky’s fixative (4% PFA, 2% glutaraldehyde, 0.1 M sodium cacodylate, pH 7.4) at least overnight at 4 **°**C. Samples were then washed with 0.1 M sodium cacodylate to remove fixative, and then post-fixed for 1 h in 2% osmium tetroxide in 0.1 M sodium cacodylate. Nerves were then dehydrated with increasing concentrations of ethanol followed by propylene oxide (PO). Samples were then infiltrated for 1–2 h in 2:1 PO:EPON, and then overnight in 1:1 PO:EPON with gentle agitation at room temperature. Samples were then transferred to 100% EPON while residual PO was allowed to fully evaporate (>4 h). For all time points, four non-overlapping images at ×1000 magnification were quantified, and all mouse TEM data is expressed per 1000 μm^2^ area. Control genotypes were *Dhh*^*Cre*(−)^*;Fbxw7*^*fl*/+^ and *Dhh*^*Cre*(−)^*;Fbxw7*^*fl*/*fl*^. For P3: *N* = 4 controls, *N* = 6 *Dhh*^*Cre*(+)^*;Fbxw7*^*fl*/+^ (Hets), and *N* = 5 *Dhh*^*Cre*(+)^*;Fbxw7*^*fl*/*fl*^ (cKO). At P21: *N* = 4 controls, *N* = 4 Hets, and *N* = 4 cKO. For P42 samples: *N* = 4 controls, *N* = 3 Hets, and *N* = 4 cKO. At 6 months of age: *N* = 5 controls, *N* = 4 Hets, and *N* = 5 cKO. For the double mutant analyses, control genotypes used were *Dhh*^*Cre*(−)^*;Fbxw7*^*fl*/+^*;mTOR*^+/+^ *Dhh*^*Cre*(−)^*;Fbxw7*^*fl*/+^*;mTOR*^*fl*/*fl*^, *Dhh*^*Cre*(−)^*;Fbxw7*^*fl*/+^*;mTOR*^*fl*/+^, *Dhh*^*Cre*(−)^*;Fbxw7*^*fl*/*fl*^*;mTOR*^*fl*/*fl*^, and *Dhh*^*Cre*(−)^*;Fbxw7*^+/+^*;mTOR*^*fl*/*fl*^. Fbxw7 “Hets” were *Dhh*^*Cre*(+)^*;Fbxw7*^*fl*/+^*;mTOR*^+/+^ littermate siblings, and “HetΔmTOR” animals were *Dhh*^*Cre*(+)^*;Fbxw7*^*fl*/+^*;mTOR*^*fl*/*fl*^ siblings. At P3: *N* = 4 controls, *N* = 6 Hets, *N* = 5 HetΔmTOR, and *N* = 1 *Dhh*^*Cre*(+)^*;Fbxw7*^*fl*/*fl*^*;mTOR*^*fl*/*fl*^ (cKOΔmTOR). For P21: *N* = 6 controls, *N* = 4 Hets, and *N* = 3 HetΔmTOR.

### Serial block-face scanning electron microscopy (SBF-SEM)

SBF-SEM was performed on a *Dhh*^*Cre*(+)^*;Fbxw7*^*fl*/*fl*^ nerve at P180 (*N* = 1; multiple regions). Nerves were fixed in 4% PFA overnight at 4 °C. Nerves were then processed for SBF-SEM by the Multiscale Microscopy Core at Oregon Health & Science University. Images were collected with 10 nm lateral resolution and 50 nm slice thickness using an FEI Teneo VolumeScope Microscope. Nerves were sectioned and imaged overnight to obtain a depth of ~50 μm. Eleven different regions of interest were imaged and analyzed across two technical experiments. Sections were annotated using FIJI, the movie was composed using Microscopy Image Browser software, and data compilation was performed using Amira. The movie shown is a representative example.

### Behavioral studies

Behavior testing was performed using mice of both sexes from 5 to 6 months of age (*N* = 11 controls, *N* = 10 cKO). The experimenter was blind to the genotypes of the mice during all data acquisition. All behavior data was analyzed using a *t*-test with Welch’s correction to determine statistical significance.

### Gross motor function (Rotarod)

An accelerating Rotarod (Ugo Basile, Comerio, Italy) as used to assess motor coordination^45^. Mice received two training sessions separated by 1 h. The first training session consisted of two trials of 120 s spent walking on the Rotarod at a fixed spped of 4 r.p.m. The second training session consisted of one trial of 120 s at 4 r.p.m. Latency to fall as the Rotarod accelerated from 4 to 40 r.p.m. over 5 min was assessed. Five consecutive experimental trials were performed with a 5 min rest interval between trials.

### Locomotor activity (open field)

Prior to testing, mice were habituated to the test room in their cages for 1 h. Locomotor activity in an open field^16^ was then assessed by recording photo beam breaks in a 42 (length) × 42 (width) × 30 (height) cm chamber for 60 min using a VersaMax Animal Activity Monitoring System (AccuScan Instruments). We then calculated the total distance traveled and the horizontal activity (beam breaks) over the entire chamber.

### Movement initiation

To assess movement initiation, we recorded the time it took each mouse to exit an 18 × 18 cm square (all four paws outside the square) marked on a flat horizontal surface.

### Complex motor function (pole test)

We used the pole test to evaluate performance of a complex motor task that requires skilled forelimb use, strength, and balance^15^. Mice were placed on a vertical metal pole that is 49 cm in height and 0.9 cm in diameter with the head of the mouse oriented upward. The time required for the mouse to turn around such that the mouse’s head is oriented downward and the hind limbs are straddling the pole was recorded. In addition, the time required for the mouse to climb down to the base of the pole was recorded.

### Cold sensitivity (acetone evaporation test)

Cold sensitivity of the hind paws was measured by applying a drop of acetone to the plantar surface of the hind paw. Five separate applications of acetone were applied to each hind paw. For each application the mouse was observed for 5 min. The percentage of applications for which the mouse responded (shaking, licking, or elevating the hind paw) to acetone application was recorded for each mouse^17^.

### Gait analysis

We used the Noldus CatWalk XT system to quantify multiple locomotor and gait parameters including: run speed, stride length, paw print area (mm^2^), maximum contact area (mm^2^), and maximum contact mean intensity (arbitrary units [a.u.]). Briefly, the mouse voluntarily traverses a meter-long glass plate and its footprints are captured by a video camera. CatWalk XT quantifies parameters related to print dimensions and gait dynamics.

### Mechanical sensitivity (von Frey)

The innocuous mechanical thresholds of both hind paws were assessed with the von Frey test. Mice were placed in plastic behavior boxes with open bottoms on a wire mesh. Varying diameter von Frey monofilaments (Stoelting, Chicago, IL) were pressed against the plantar surface of the hind paw until the filament bent. The force applied to the hind paw is dependent on the diameter of the filament. The up/down method described by Chaplan was used to determine the withdrawal threshold^46^.

### Heat sensitivity (Hargreaves)

Heat sensitivity was evaluated by using a paw thermal stimulation system in which a source of radiant heat (active intensity = 15) was applied to the plantar surface of the hind paw and the paw withdrawal latency was measured^47^. We performed three trials on each paw. The withdrawal latencies obtained in each of the six trials were averaged to obtain the withdrawal latency for each mouse.

### Myelinating Schwann cell cultures

Dissociated SC/dorsal root ganglia (DRG) sensory neuron co-cultures were prepared as previously described^48–50^. Briefly, DRGs were isolated from individual embryonic day 13.5 (E13.5) *Dhh*^*Cre*(−)^*;Fbxw7*^*fl*/*fl*^ (control) or *Dhh*^*Cre*(+)^*;Fbxw7*^*fl*/*fl*^ (cKO) mouse littermate embryos. DRGs were washed with L15 medium and then incubated in 0.25% trypsin at 37 **°**C for 30 min. Trypsin was removed, and DRGs were washed with L15 + 10% FBS and centrifuged gently at 1000 r.p.m. for 10 min. The medium was replaced with DRG medium (high glucose MEM with 10% FBS and 100 ng/mL NGF), and DRGs were triturated with a fire-polished Pasteur pipette until homogenous. The suspension was plated at 150,000 cells in the center of a collagen-coated 25 mm coverslip. Cultures were maintained in DRG medium for 5–6 days, after which 50 µg/mL ascorbic acid was added to the medium to induce myelination. Cultures were fixed in 4% PFA for 15 min after ten days in media containing ascorbic acid. Cultures were blocked and permeabilized with 20% normal goat serum (NGS) and 0.2% Triton X-100 in PBS for 1 h at room temperature, and then incubated overnight at 4 °C with the following antibodies in 20% NGS: mouse anti-neurofilament medium chain (1:200; Millipore), rat monoclonal anti-MBP (1:100; Millipore), and rabbit anti-Krox20 (1:500; provided by Dies Meijer). Cultures were then incubated with Alexa Fluor AffiniPure goat anti-mouse 488 (1:1000), AffiniPure goat anti-rat 594 (1:1000), and goat anti-rabbit 647 (1:500) for 1 h at room temperature, counterstained with DAPI, and dried before mounting in Prolong Gold Mountant (Invitrogen). Cultures were imaged as z-stacks using a ×40 oil 1.3NA objective on a Zeiss AxioImager with ApoTome. Entire cultures were imaged and analyzed by an experimenter blinded to genotype, and counts represent the number of multipolar MBP(+)/Krox20(+) SCs per culture. Data represent two technical replicate cultures from each of three independent mouse embryos per genotype.

### Western blot analyses

To assess mTOR protein levels in the sciatic nerve, we dissected nerves from the sciatic notch to just proximal to the trifurcation. These nerve segments were flash-frozen in liquid nitrogen, cut into small pieces with microdissection scissors, and homogenized in lysis buffer (20 mm Tris-HCl, pH 7.5, 150 mm NaCl, 1 mm Na_2_EDTA, 1 mm EGTA, 1% Triton X-100, 2.5 mm sodium pyrophosphate, 1 mm β-glycerophosphate, 1 mm Na_**3**_VO_**4**_, 1 μg/ml leupeptin) with phosphatase inhibitor mixtures 1 and 2 (Invitrogen, ThermoFisher Scientific, Waltham, MA). Equal protein amounts (15 μg) were loaded and analyzed by SDS-PAGE and western blot. Antibodies used were anti-mTOR (1:1000; Cell Signaling Technologies, Danvers, MA) and anti-α-tubulin (1:1000; Abcam, Cambridge, MA). Western blot images were quantified using FIJI. All bands were normalized to background and mTOR bands were compared with α-tubulin levels.

### RNA isolation and reverse transcription

Total RNA was extracted from flash-frozen P21 mouse sciatic nerves (*N* = 3 *Dhh*^*Cre*(−)^*;Fbxw7*^*fl*/*fl*^ littermate controls and *N* = 3 *Dhh*^*Cre*(+)^*;Fbxw7*^*fl*/*fl*^ animals), using a standard TRIzol extraction protocol (Life Technologies, ThermoFisher Scientific, Waltham, MA). Briefly, TRIzol was added to the frozen tissue samples, which were then allowed to thaw at room temperature for 10 min. During this incubation time, and while still in TRIzol, nerves were cut into much smaller pieces using microdissection scissors. Samples were homogenized via disruption with a plastic-tipped electric homogenizer, followed by passage through a syringe and 22.5 g needle at least ten times, and then a 27 g needle at least ten more times until no lumps of tissue were observed. Once the nerves had been homogenized, we proceeded as usual with the standard TRIzol RNA extraction procedure as per manufacturer instructions.

Total RNA (500 ng) was then reverse transcribed in 20 μl using the Superscript III First Strand cDNA Synthesis Kit (Invitrogen, ThermoFisher Scientific, Waltham, MA) using random hexamers, as per manufacturer instructions. All cDNA products were diluted 1:5 prior to use in qPCR reactions.

### Quantitative reverse transcription PCR

To assay mRNA expression levels of mTOR and members of the mTOR signaling pathway, we used the RT^2^ Profiler PCR Array for Mouse mTOR Signaling (Qiagen, PAMM-098ZA, Valencia, CA). A complete gene list can be found on the manufacturer’s website. All assays were performed on a ViiA7 Real-Time PCR system (Applied Biosystems, ThermoFisher Scientific, Waltham, MA), in a total volume of 10 μl using 2X SsoFast Evagreen Supermix (BioRad, Hercules, CA) and 50 ng of cDNA per reaction. Standard qPCR settings were used: 95 °C for 10 min followed by 40 cycles of 95 °C for 15 s (sec) then 60 °C for 30 s, followed by melt curve analysis. As suggested by the RT^2^ profiler manual, we adjusted the ramp rate to 1 °C/s. All controls including housekeeping genes, positive controls for amplification, and controls for genomic DNA contamination were included as standards in the array.

qPCR data were analyzed using Microsoft Excel. Relative expression was calculated using the ΔΔCt method^51^. Genomic contamination was negligible in all samples. To control for input variations, ΔCt was calculated by comparing the Ct of each gene of interest (GOI) to the average Ct of the five housekeeping genes (*Actb*, *B2m*, *Gapdh*, *Gusb*, and *Hsp90ab1*) for that sample. ΔΔCt was then calculated relative to expression compared with that seen in the littermate control. Average relative expression (RQ), or fold change (2−ΔΔCt), over controls is shown in Fig. S3. All error bars depict RQmax and RQmin, which represent the maximum and minimum limits of possible RQ values based on the standard error of the ΔCt values. The gray line at *y* = 1 represents the controls.

### RNA sequencing data mining

To analyze the expression of *Fbxw7, mTOR*, and other Fbxw7 targets, we mined previously reported RNA sequencing data^24^ from wild-type sciatic nerves at P3 and P21. We generated a list of candidates through a series of basic literature searches focusing primarily on targets of Fbxw7 that might have a role in SCs (c-Jun, Notch, Myc, Ccne1 (cyclinE), PGC1, SREBP1, and GATA1). This list is not intended to represent an exhaustive list of Fbxw7 targets. Using Excel, we searched the raw data for the gene names of *Fbxw7*, *mTOR*, and our candidate targets. We then recorded the raw FPKM value for each animal at each time point (*N* = 4 for P3 and *N* = 3 for P21) and transferred that information into Graphpad PRISM for analysis. We then averaged the FPKM values for each time point, calculated the standard deviation (S.D.; error bars shown), and generated the graph shown in Fig. 6a using PRISM 8 for MacOS.

### Immunofluorescence

Sciatic nerves were isolated and fixed in 4% paraformaldehyde (PFA) overnight at 4 °C. After washing with PBS and 30% sucrose, nerves were embedded in OCT and frozen at −80 °C. Cryo-sections were acquired in cross-section orientation at 15 μm thickness. Slices were brought to room temperature and then incubated with blocking solution (2% bovine serum albumin, 2% normal goat serum, 0.2% Triton in 1× PBS). The following primary antibodies were diluted in blocking solution and incubated for 2 h at room temperature: Anti-S100 beta [EP1576Y] (Alexa Fluor 488—Abcam 1:200) and Anti-c-Jun (60A8, Cell Signaling 1:200). Samples were then washed three times (5 min each) in 1× PBS, and slides were incubated with secondary species-specific Invitrogen antibodies for 1 h or mounted using Vectashield with DAPI (Vector Labs) to label nuclei. Fluorescent images were obtained with a Zeiss AxioImager microscope. CZI files were analyzed using FIJI. All data were quantified blindly. *N* = 3 per genotype. c-Jun data are shown in Fig. 6.

### Quantification and statistical analysis

All data are reported as mean + standard deviation (S.D.). Statistically significant differences were determined using one-way ANOVA for all experiments with more than two groups but only one dependent variable. Similarly, two-way ANOVA was used for experiments with multiple groups and two dependent variables. All experiments with only two groups and one dependent variable were compared using an unpaired *t*-test with Welch’s correction, which assumes unequal variance. Figure legends specify which test was used for specific experiments. In all cases, * = *p* < 0.05; ** = *p* < 0.01; *** = *p* < 0.001; and **** = *p* < 0.0001; NS = not significant. In all cases, asterisks immediately above a bar indicate the significance of that sample relative to the control sample. If any other comparisons, such as *Dhh*^*Cre*(+)^*;Fbxw7*^*fl*/+^ (Het) to *Dhh*^*Cre*(+)^*;Fbxw7*^*fl*/*fl*^ (cKO), were significant, this is indicated with a bar spanning above the two samples being compared with the appropriate asterisks. If not indicated otherwise, the comparison was not significant. In most cases, *Dhh*^*Cre*(+)^*;Fbxw7*^*fl*/+^ samples were not statistically distinguishable from *Dhh*^*Cre*(+)^*;Fbxw7*^*fl*/*fl*^.

### Reporting summary

Further information on research design is available in the Nature Research Reporting Summary linked to this article.

## Supplementary information

Supplementary Information

Peer Review

Reporting Summary

Description of Additional Supplementary Files

Supplementary Movie 1

## Data Availability

No new genomic datasets were generated or analyzed during the current study. Raw data from the SBF-SEM analysis will be made freely available upon request.
