# Supplementary material for: Myelinating Schwann cells ensheath multiple axons in the absence of E3 ligase component Fbxw7
Source: Nat Commun. 2019 Jul 5;10:2976. doi: 10.1038/s41467-019-10881-y (PMC6611888; doi:10.1038/s41467-019-10881-y)
Supplement: Supplementary file 1 — Supplementary Information [file 41467_2019_10881_MOESM1_ESM.pdf]

Supplementary Information for:

Myelinating Schwann cells ensheath multiple axons in the absence of E3 ligase component Fbxw7

Harty et al.

SUPPLEMENTARY FIGURE 1

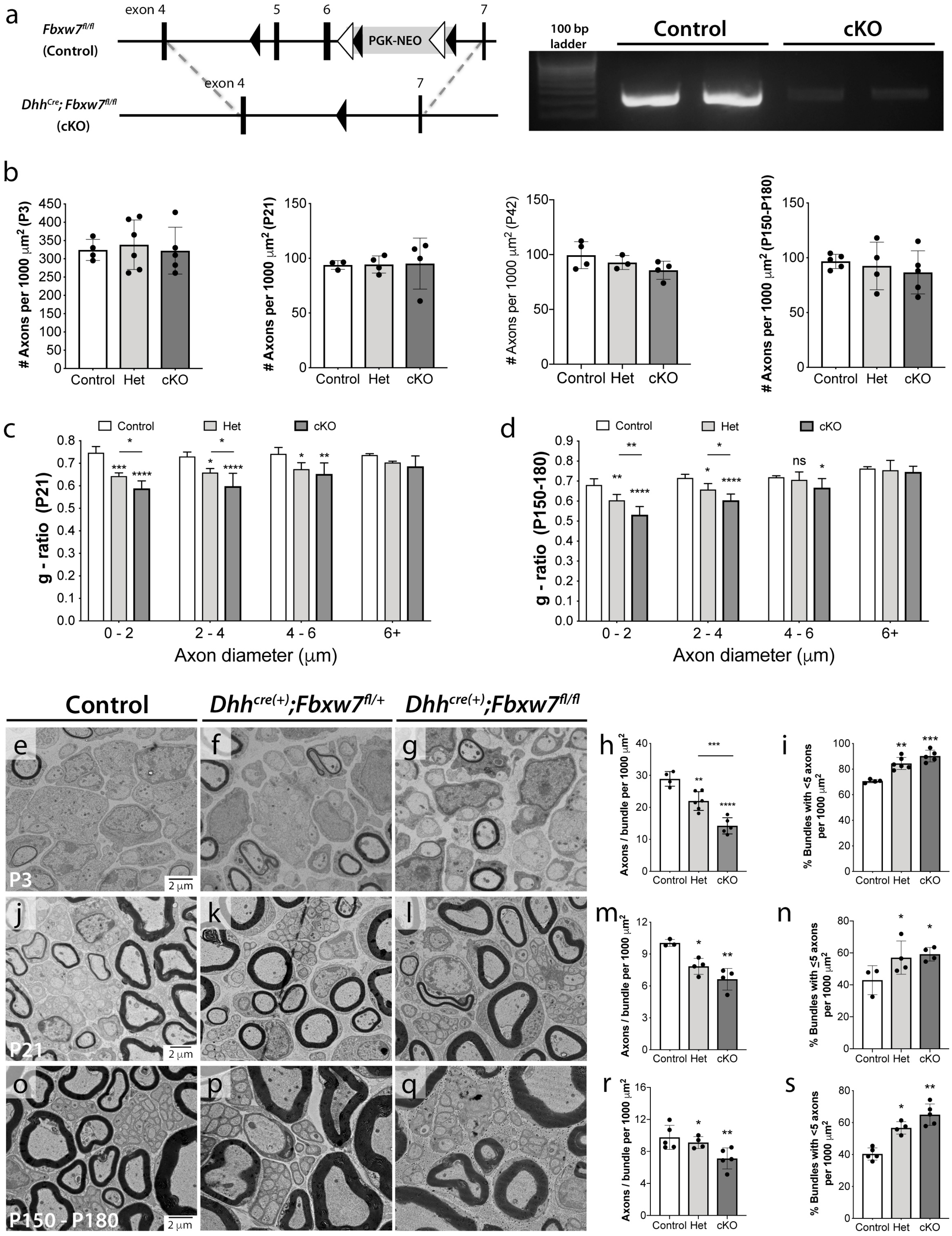

Supplementary Figure 1: Fbxw7 cell-autonomously regulates SC radial sorting and Remak bundle ensheathment. (a) Exons 5 and 6 are targeted in *Fbxw7* in *Dhh*$^{cre(+)}$*:Fbxw7*$^{fl/fl}$ animals. RT-PCR shows that *Fbxw7* mRNA is significantly reduced in sciatic nerves of *Dhh*$^{cre(+)}$*/Fbxw7*$^{fl/fl}$ (cKO) compared to *Dhh*$^{cre(-)}$*/Fbxw7*$^{fl/fl}$ (control) at P21. (b) Total axon number is not statistically different across genotypes at every stage assessed (p>0.05 for all comparisons). (c-d) Myelin thickness is increased, particularly on small axons, in *Fbxw7* mutant nerves beginning at P21 (c; 0-2 µm: Control to Het p=0.0004, Control to cKO p<0.0001, Het to cKO p=0.0355; 2-4 µm: Control to Het p=0.0149, Control to cKO p<0.0001, Het to cKO p=0.0183; 4-6 µm: Control to Het p=0.0227, Control to cKO p=0.0020, Het to cKO p=0.5585; two way ANOVA) and persisting until at least P180 (d; 0-2 µm: Control to Het p=0.0023, Control to cKO p<0.0001, Het to cKO p=0.0045; 2-4 µm: Control to Het p=0.0262, Control to cKO p<0.0001, Het to cKO p=0.0398; 4-6 µm: Control to Het p=0.8195, Control to cKO p=0.0344, Het to cKO p=0.1704; two way ANOVA). (e-i) TEM at P3 shows that loss of *Fbxw7* increases segregation of axons during radial sorting (axons/bundle: h; Control to Het p=0.0042, Control to cKO p<0.0001, Het to cKO p=0.0011; one way ANOVA), (percentage of bundles with≤5 axons: i; Control to Het p=0.0053, Control to cKO p=0.0005, Het to cKO p=0.2628; one way ANOVA). This phenotype persists at P21 (j-n; m: Control to Het p=0.0321, Control to cKO p=0.0034, Het to cKO p=0.2369; n: Control to Het p=0.0412, Control to cKO p=0.0462, Het to cKO p=0.9889; one way ANOVA) through at least 6 months of age (P150-P180; o-s; r: Control to Het p=0.0280, Control to cKO p=0.0019, Het to cKO p=0.3909; s: Control to Het p=0.0363, Control to cKO p=0.0010, Het to cKO p=0.1821; one way ANOVA). ns=not significant. Asterisks above bars indicate comparisons to controls. Unless otherwise indicated, comparisons between *Dhh*$^{cre(+)}$*;Fbxw7*$^{fl/+}$ and *Dhh*$^{cre(+)}$*;Fbxw7*$^{fl/fl}$ were not significant. P3: N=4 control, 6 heterozygous, 5 cKO. P21: N=3 control, 4 heterozygous, 4 cKO. P150-180: N=5 control, 4 heterozygous, 5 cKO. Error bars depict SD.

SUPPLEMENTARY FIGURE 2

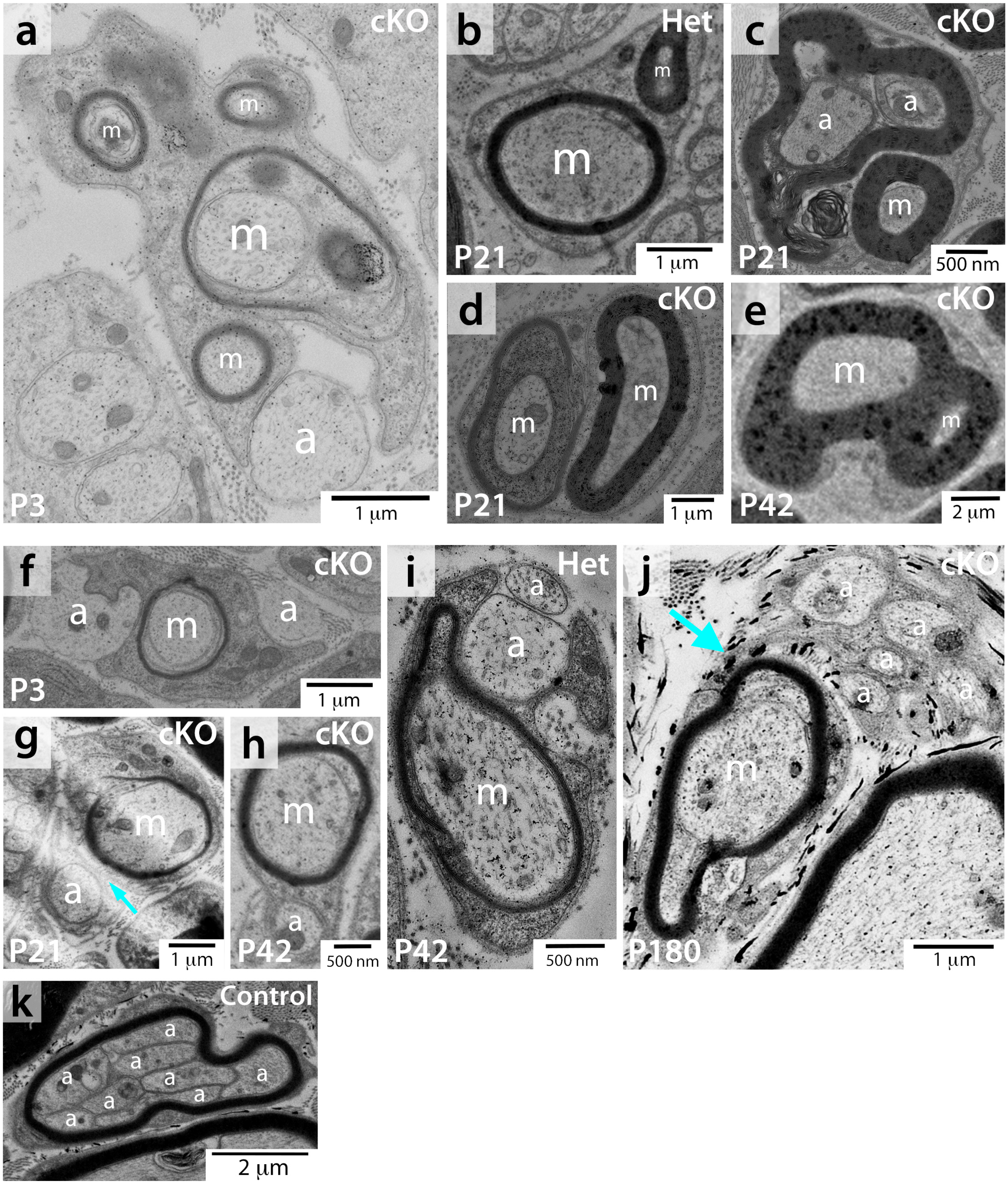

Supplementary Figure 2: Enhanced myelinating potential in *Fbxw7* mutant SCs. Additional examples of apparent multi-axonal ensheathment by myelinating SCs in Het and cKO animals at each time point – P3 (a, f), P21 (b-d, g), P42 (e, h, i) and P150-180 (j). *Fbxw7* mutant SCs were occasionally seen appearing to myelinate as many as four independent axons (a; myelinated axons indicated by m; unmyelinated axons indicated by a). (a, f-j) Additional examples of SCs that appear to have both myelinated larger axons as well as encompassed several non-myelinated axons like myelinating/Remak SC hybrids – P3 (a, f), P21 (g), P42 (e, h, i) and P150-180 (j). Here again, the myelinated axon and the bundle of unmyelinated axons were sometimes linked by thin processes of SC cytoplasm (g, j; blue arrows). These phenotypes are distinct from polyaxonal myelination in which a bundle of small-diameter axons is myelinated as though the bundle were a single large axon (k).

SUPPLEMENTARY FIGURE 3

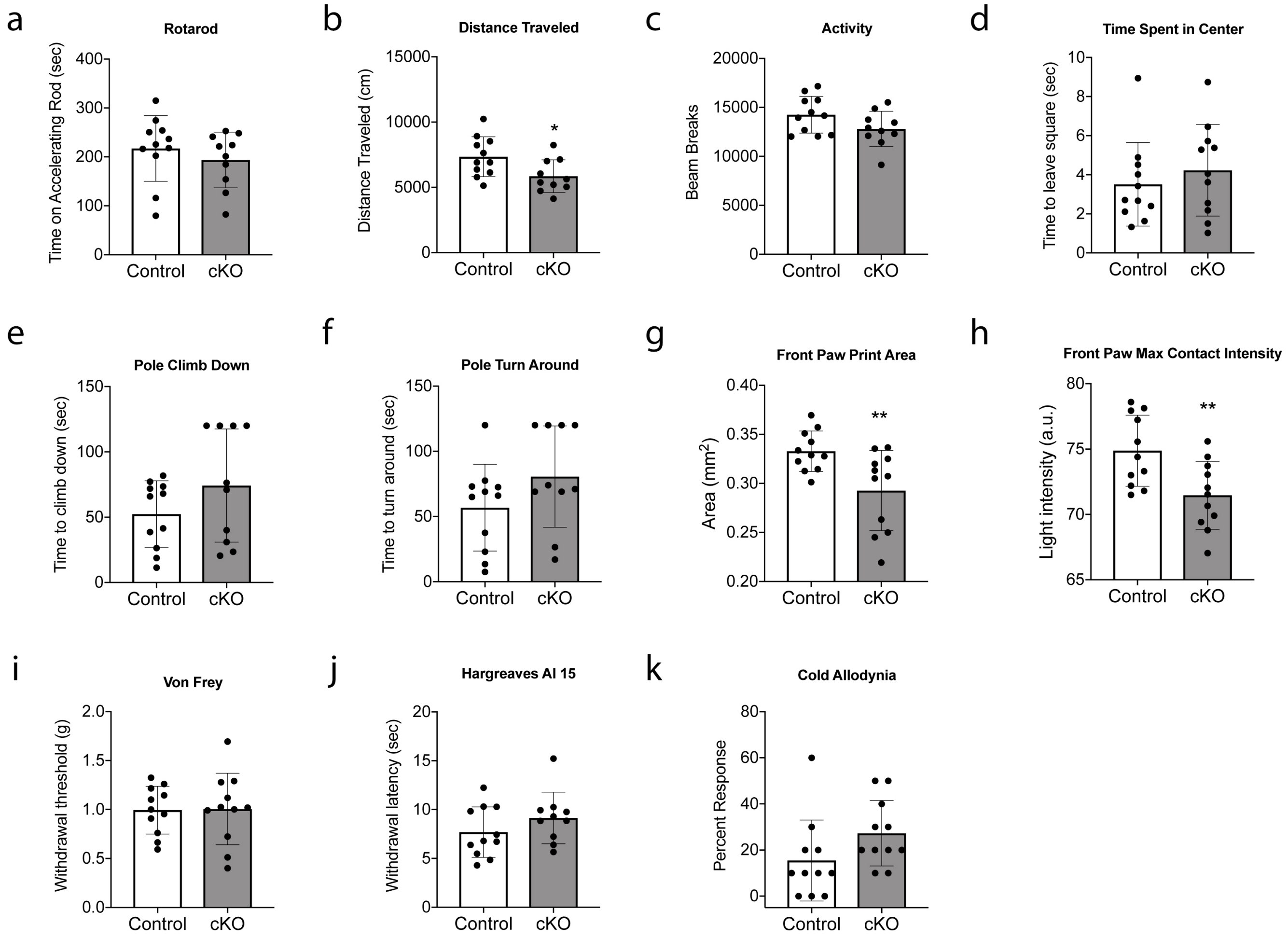

Supplementary Figure 3: Loss of *Fbxw7* causes mild motor and sensory deficits. A battery of sensory and motor tests (a-k) showed that at 5-6 months, loss of *Fbxw7* results in mild behavioral changes. Motor tests included: accelerating rotarod (a), open field (b,c; b – p=0.0253), movement initiation (d), the pole test (e,f) and gait analyses (g,h; g – p=0.0091; h – p=0.0070). Mice were also tested for sensitivity to mechanical stimuli (i), heat (j), and cold (k). Unpaired *t*-test with Welch's correction, N = 11 controls, N = 10 cKO (conditional knockout). Unless otherwise indicated the comparison is not significant (p>0.05). AI, active intensity. Error bars are SD.

SUPPLEMENTARY FIGURE 4

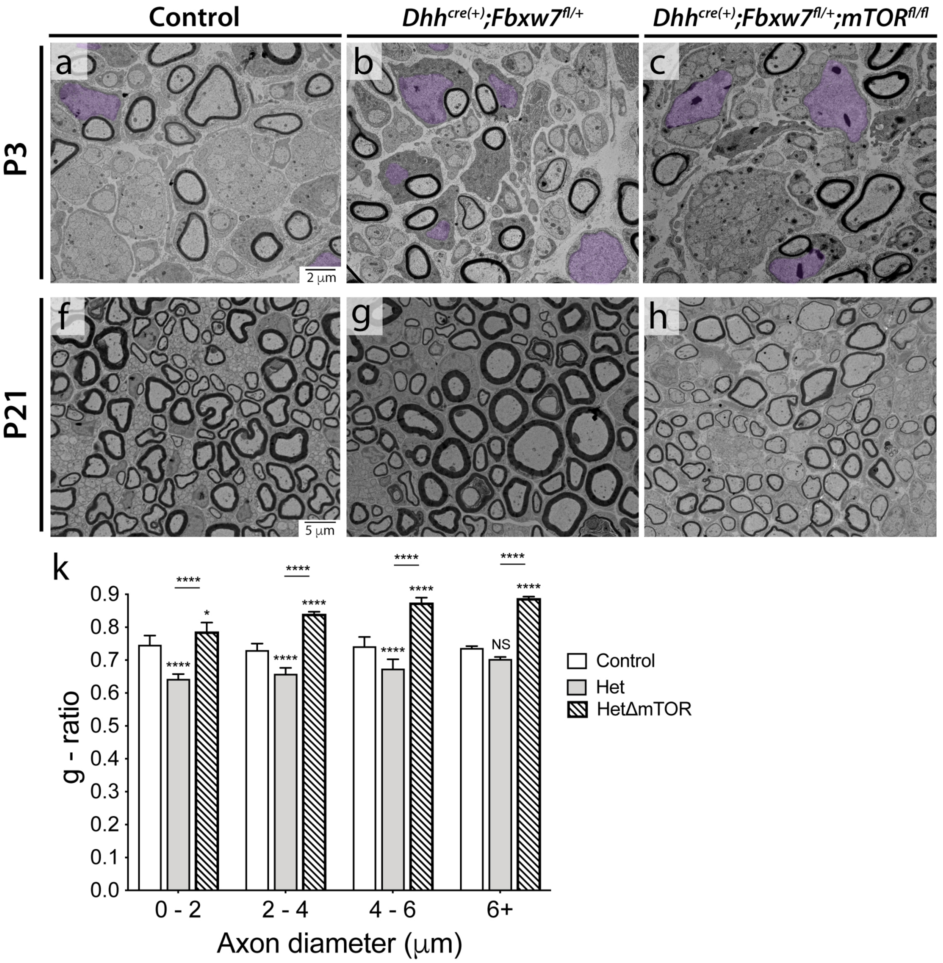

Supplementary Figure 4: Genetic inhibition of mTOR suppresses the SC number, radial sorting, and myelin thickness phenotypes seen in Fbxw7 mutant nerves. (a-j) Transmission electron micrographs (TEMs) of SC-specific double mutant *Fbxw7:mTOR* mice at P3 (a-e) and P21 (f-j) demonstrates that $Dhh^{cre(+)};Fbxw7^{fl//+};mTOR^{+/+}$ (b; Het [heterozygous]) nerves display greater numbers of SC nuclei as well as a higher percentage of myelinated axons relative to controls (a). Loss of mTOR in $Dhh^{cre(+)};Fbxw7^{fl/+};mTOR^{fl/fl}$ (HetΔmTOR; c) nerves suppresses both of these phenotypes (d,e; d - Control to Het p=0.0020, Control to HetΔmTOR p=0.1232, Het to HetΔmTOR p<0.0001; e - Control to Het p=0.0037, Control to HetΔmTOR p=0.0643, Het to HetΔmTOR p<0.0001; one way ANOVA). Deletion of *mTOR* further suppressed the radial sorting and Remak SC ensheathment defects observed in $Dhh^{cre(+)};Fbxw7^{fl//+};mTOR^{+/+}$ nerves (f-j; i - Control to Het p=0.0360, Control to HetΔmTOR p=0.0033, Het to HetΔmTOR p=0.0002; j - Control to Het p=0.0317, Control to HetΔmTOR p=0.1752, Het to HetΔmTOR p=0.0037; one way ANOVA). $Dhh^{cre(+)};Fbxw7^{fl/+};mTOR^{fl/fl}$ sciatic nerves also displayed markedly thinner myelin (increased g-ratios) relative to both control and $Dhh^{cre(+)};Fbxw7^{fl//+};mTOR^{+/+}$ siblings (k; 0-2 µm: Control to Het p<0.0001, Control to HetΔmTOR p=0.0364, Het to HetΔmTOR p<0.0001; 2-4 µm: Control to Het p<0.0001, Control to HetΔmTOR p<0.0001, Het to HetΔmTOR p<0.0001; 4-6 µm: Control to Het p<0.0001, Control to HetΔmTOR p<0.0001, Het to HetΔmTOR p<0.0001; 6+ µm: Control to Het p=0.0541, Control to HetΔmTOR p<0.0001, Het to HetΔmTOR p<0.0001; two way ANOVA). P3: N = 4 controls, N = 6 $Dhh^{cre(+)};Fbxw7^{fl//+};mTOR^{+/+}$, N = 5 $Dhh^{cre(+)};Fbxw7^{fl/+};mTOR^{fl/fl}$. For P21: N = 6 controls, N = 4 $Dhh^{cre(+)};Fbxw7^{fl//+};mTOR^{+/+}$, and N = 3 $Dhh^{cre(+)};Fbxw7^{fl/+};mTOR^{fl/fl}$. Error bars depict SD.
