# Supplementary material for: Myelinating Schwann cells ensheath multiple axons in the absence of E3 ligase component Fbxw7
Source: Nat Commun. 2019 Jul 5;10:2976. doi: 10.1038/s41467-019-10881-y (PMC6611888; doi:10.1038/s41467-019-10881-y)
Supplement: Supplementary file 3 — Reporting Summary [file 41467_2019_10881_MOESM3_ESM.pdf]

nature research

Corresponding author(s): Kelly R. Monk

Last updated by author(s): May 22, 2019

Reporting Summary

Nature Research wishes to improve the reproducibility of the work that we publish. This form provides structure for consistency and transparency in reporting. For further information on Nature Research policies, see Authors & Referees and the Editorial Policy Checklist.

Statistics

For all statistical analyses, confirm that the following items are present in the figure legend, table legend, main text, or Methods section.

n/a	Confirmed	
☐	☒	The exact sample size (*n*) for each experimental group/condition, given as a discrete number and unit of measurement
☐	☒	A statement on whether measurements were taken from distinct samples or whether the same sample was measured repeatedly
☐	☒	The statistical test(s) used AND whether they are one- or two-sided<br>*Only common tests should be described solely by name; describe more complex techniques in the Methods section.*
☐	☒	A description of all covariates tested
☐	☒	A description of any assumptions or corrections, such as tests of normality and adjustment for multiple comparisons
☐	☒	A full description of the statistical parameters including central tendency (e.g. means) or other basic estimates (e.g. regression coefficient) AND variation (e.g. standard deviation) or associated estimates of uncertainty (e.g. confidence intervals)
☒	☐	For null hypothesis testing, the test statistic (e.g. *F*, *t*, *r*) with confidence intervals, effect sizes, degrees of freedom and *P* value noted<br>*Give P values as exact values whenever suitable.*
☒	☐	For Bayesian analysis, information on the choice of priors and Markov chain Monte Carlo settings
☒	☐	For hierarchical and complex designs, identification of the appropriate level for tests and full reporting of outcomes
☒	☐	Estimates of effect sizes (e.g. Cohen's *d*, Pearson's *r*), indicating how they were calculated

Our web collection on statistics for biologists contains articles on many of the points above.

Software and code

Policy information about availability of computer code

Data collection	The Noldus CatWalk XT System was used to collect data on gait. Zeiss ZEN software was used to collect immunofluorescence data.
Data analysis	Data was analyzed using Microsoft Excel, PRISM, FIJI, and Microscopy Image Browser.

For manuscripts utilizing custom algorithms or software that are central to the research but not yet described in published literature, software must be made available to editors/reviewers. We strongly encourage code deposition in a community repository (e.g. GitHub). See the Nature Research guidelines for submitting code & software for further information.

Data

Policy information about availability of data

All manuscripts must include a data availability statement. This statement should provide the following information, where applicable:

- Accession codes, unique identifiers, or web links for publicly available datasets
- A list of figures that have associated raw data
- A description of any restrictions on data availability

Raw data for all figures can be made available upon request.

Field-specific reporting

Please select the one below that is the best fit for your research. If you are not sure, read the appropriate sections before making your selection.

☒ Life sciences ☐ Behavioural & social sciences ☐ Ecological, evolutionary & environmental sciences

For a reference copy of the document with all sections, see nature.com/documents/nr-reporting-summary-flat.pdf

Life sciences study design

All studies must disclose on these points even when the disclosure is negative.

Sample size	In every experiment except the functional assessments and the 3D EM, we used Ns of 3-5 mice per genotype, which is standard in the field when using mice with pure backgrounds. For functional assessments we used Ns of 10-11 mice per genotype because greater sample size is required for behavioral studies. For 3D EM, we performed two technical replicates on a single mutant nerve because this experiment primarily served as secondary support for other data collected with greater sample size and 3D EM is both technically challenging and costly.
Data exclusions	No data were excluded from this study.
Replication	Each experiment presented in the manuscript was repeated in multiple animals. All results in the study are drawn from the analysis of multiple animals (with the exception of the 3D EM experiment) and were replicated from animal to animal. When possible (immunofluorescence, western blot) multiple individuals independently performed the same experiment and we verified that the results were consistent from experiment to experiment and experimenter to experimenter.
Randomization	Mice were allocated into groups based on genotype. To control for possible background differences, we analyzed littermate controls and only used mice with pure C57Bl/6 background.
Blinding	In all cases except 3D EM, experimenters were blind to genotype during data collection and analysis. In the case of the 3D EM experiment, the experimenter was not blind to genotype because we only analyzed a single nerve per genotype. Since the experimenter was aware of the genotype and the sample size was N=1, the 3D EM data were not quantified.

Reporting for specific materials, systems and methods

We require information from authors about some types of materials, experimental systems and methods used in many studies. Here, indicate whether each material, system or method listed is relevant to your study. If you are not sure if a list item applies to your research, read the appropriate section before selecting a response.

Materials & experimental systems

n/a	Involved in the study	
☐	☒	Antibodies
☒	☐	Eukaryotic cell lines
☒	☐	Palaeontology
☐	☒	Animals and other organisms
☒	☐	Human research participants
☒	☐	Clinical data

Methods

n/a	Involved in the study	
☒	☐	ChIP-seq
☒	☐	Flow cytometry
☒	☐	MRI-based neuroimaging

Antibodies

Antibodies used	Antibodies used in this study include mouse anti-Neurofilament medium chain (Millipore), rat monoclonal anti-MBP (Millipore), rabbit anti-Krox20 (provided by Dies Meijer), anti-mTOR (Cell Signaling Technologies), and anti-alpha-tubulin (Abcam).
Validation	Anti-Neurofilament medium chain and rat monoclonal anti-MBP have been validated by the manufacturer for immunofluorescence studies in mouse tissue. The anti-Krox20 antibody validation data for immunofluorescence in mice can be found in Darbas et al. (2004). Both anti-mTOR and anti-alpha-tubulin have been validated for use in western blot applications in mice by their respective manufacturers.

Animals and other organisms

Policy information about studies involving animals; ARRIVE guidelines recommended for reporting animal research

Laboratory animals	We used the C57Bl/6 strain of mice (Mus musculus) at postnatal days 3, 21, 42, and 150-180. Mice of both sexes were analyzed.
Wild animals	This study did not involve wild animals.
Field-collected samples	This study did not involve field-collected samples.
Ethics oversight	Animal use and ethical protocols were approved by the Institutional Animal Care and Use Committees at Washington University School of Medicine (St. Louis, MO), Oregon Health & Science University (Portland, OR), and UCSF (San Francisco, CA).

Note that full information on the approval of the study protocol must also be provided in the manuscript.
