# Supplementary material for: Myelinating Schwann cells ensheath multiple axons in the absence of E3 ligase component Fbxw7
Source: Nat Commun. 2019 Jul 5;10:2976. doi: 10.1038/s41467-019-10881-y (PMC6611888; doi:10.1038/s41467-019-10881-y)
Supplement: Supplementary file 4 — Description of Additional Supplementary Files [file 41467_2019_10881_MOESM4_ESM.docx]

**Description of Additional Supplementary Files**

File name: Supplementary Movie 1: 3D Imaging of *Fbxw7* mutant nerves.

Description: Serial block-face scanning electron microscopy was used to image an *Fbxw7* mutant nerve at P180. 11 different regions of interest were analyzed; the cell featured in this movie is a representative example. The cell pictured is seen extending a cytoplasmic process between an unmyelinated axon (bottom; axon marked with a) and a myelinated axon (top; myelinated marked with m). Although in some sections the SC cytoplasm around these axons is clearly not connected, in several other sections, the cytoplasm and cell membrane are observed to be continuous between the unconnected sections. Images were collected with 10 nm lateral resolution and 50 nm slice thickness. This movie represents approximately 50 μm of nerve depth. Scale bar = 2μm.
