# Supplementary material for: Myelinating Schwann cells ensheath multiple axons in the absence of E3 ligase component Fbxw7
Source: Nat Commun. 2019 Jul 5;10:2976. doi: 10.1038/s41467-019-10881-y (PMC6611888; doi:10.1038/s41467-019-10881-y)
Supplement: Supplementary file 2 — Peer Review [file 41467_2019_10881_MOESM2_ESM.pdf]

Editorial Note: This manuscript has been previously reviewed at another journal that is not operating a transparent peer review scheme. This document only contains reviewer comments and rebuttal letters for versions considered at *Nature Communications.*

Reviewers' Comments:

Reviewer #1:
Remarks to the Author:
I have seen a prior version of this paper before for another journal and maintain that this is a very well presented study. It reports a hypermyelination phenotype of the PNS in zebrafish caused by a mutation in the gene for FBXW7, which is then more systematically studied in mice with the conditional gene targeting of Schwann cells. FBXW7 is member of the E3 ubiquitin ligase complex and known to have mTOR as a downstream target. The CNS phenotype of these mutants was previously published. Here, the paper concentrates on the description of elevated mTOR signaling in Schwann cells, thereby confirming similar observations in mouse mutants with loss of Pten or increased Akt expression. The authors also report a few novel features, such as multi-axonal myelination profiles. However, the latter appears not be caused by mTOR activation, as heterozygosity of mTOR in conditional Fbxw7 double mutants does not interfere with these phenotypes. What other targets of FBXW7 are responsible remains unknown.

I also repeat one critical comment that while the paper is nicely written and provides very beautiful data, the question comes up what is really new and could not be predicted from the known facts that (a) FBXW7 is required to degrade mTOR and (b) abnormally high mTOR signaling causes the hypermyelination phenotype detailed in this and other manuscripts. I am still missing some analysis of FBXW7 itself as a Schwann cell protein and its interaction with relevant target proteins. When and where is it expressed? Is the FBXW7 function itself regulated, e.g. in Schwann cell development, or is the protein constitutively active in the lineage? What are the (other) targets of FBXW7-dependent ubiquitination in Schwann cells?

Several other minor comments that I had previously made are all taken care of in this extensively revised manuscript.

Reviewer #2:
Remarks to the Author:
The manuscript by Harty et al., attempts to demonstrate a novel role for Fbxw7 as a important regulator of Schwann cell myelinating potential. Deletion of Fbxw7 results in the myelination of multiple axons and a hypermyelination phenotype. The authors demonstrate that mTOR dysregulation underlies the hypermyelination phenotype, however the ability of SCs to myelinate multiple axons was independent of mTOR activation. This is a fascinating study and one that speaks to the intrinsic differences between oligodendrocytes and SCs. The experiments are well detailed and rigorous. However, there are just a few suggestions that should be addressed to help make the conclusions even more compelling.

1. The authors propose that Fbxw7 is an upstream suppressor of mTOR expression/activation and that this is likely the result of Akt. Can the authors provide p-Akt levels from the sciatic nerve lysates? Additionally, the Western blot of mTOR is a bit saturated and the only observable differences are based on the decrease in the tub loading control. It would benefit the rigor of the study to show multiple lanes of sciatic nerve samples and maybe include additional control proteins like various myelin proteins, Krox20 and maybe even Oct6.
2. The title of the manuscript does not fully convey the data presented nor the novelty of the study. Fbxw7 acts as a suppressor of mTOR expression activation and mediates proper myelin deposition/thickness. Additionally, it ensures that SCs myelinate a single axon. As it stands, the title does not describe the surprising findings in the manuscript.
3. As axonal signals are especially important for the induction of myelination, what axonal signals contribute to Fbxw7 activation/expression to achieve the proper g-ratios? If the Fbxw7 SCs resemble SCs after injury, what extrinsic cues contribute to this conversion? Additional discussion on these

topics would be helpful.

Reviewer #3:
Remarks to the Author:
In their manuscript "Fbxw7 is a critical regulator of Schwann cell myelinating potential" the authors describe a novel role for the gene Fbxw7 in regulating myelination by Schwann cells (SCs). Fbw7 mutants display an increased number of SCs, a higher proportion of myelinated axons during early development and thicker myelin sheaths. The authors show that these hypermyelination phenotypes are due to the dysregulation of mTOR pathway. Intriguingly, Fbw7 mutants exhibit additional mTOR-independent phenotypes that the authors interpret as SCs myelinating multiple axons or forming both a myelin sheath and a Remak bundle, with abnormal cytoplasmic processes connecting these different sheaths. If true it would suggest that loss of Fbxw7 causes SCs to adopt an oligodendrocyte-like morphology and function, and completely novel phenotype. However, I have some concerns about the interpretation of the phenotype and think that additional work would strengthen this very interesting manuscript.

Major points:

Phenotype 1: Multiple axons myelinated.
I am not entirely convinced that Fbxw7 causes SCs to myelinate multiple axons. In the serial EM sections presented as Supplemental Movie 1, the authors label a SC that they interpret as myelinating two axons (starting at first frame). The labelled SC seems to contain two axons from the first frame, but, the deeper sections reveal that the bottom right myelin sheath closes in on itself and then disappears. If this was an axon myelinated by the SC, the deeper sections would show unmyelinated segments of that axon. Three-dimensionally, my interpretation of this sheath that closes in on itself as you section deeper is that this was a "dome" or "sphere" of myelin and not a normal cylindrical myelin sheath around an intact axon. This phenotype seems likely to be a result of extreme myelin outfolding/infolding, similar to the range of phenotypes described before for other regulators of the PTEN/Akt/mTor pathways and clinically in Charcot Marie Tooth disease and various peripheral neuropathies. See for example Sander et al. and Demonech-Estevez et al. (citations below).

Indeed, following other Schwann cells through the movie, we see additional examples of complex myelin outfoldings that seem to appear and disappear as we traverse the length of the nerve: e.g. the SC on the bottom left that gets highlighted with yellow arrows has another large "myelin dome", visible from beginning of movie to ~4s when it connects to the main sheath; it also has a "myelin sphere" visible from 9s to 12s.

This is certainly an interesting phenotype, but I do not think it is fair to conclude that Fbxw7 Schwann cells make multiple myelin sheaths. All of the multiple myelin sheaths seen in single section TEM micrographs in the figures/quantification could just as easily be due to catching a complex outfolding in a section where it appears to be multiple axons. Perhaps the clearest and easiest way to see if these mutant Schwann cells really do myelinate multiple axons and the extent to which this happens would be to sparse label them (genetically, virally, or possibly in a teased nerve preparation) and visualize using light microscopy.

Also, is there any evidence of axon loss in these mutants? Is it possible that myelin around a structure that appears axonal but does not extend cylindrically (as in the movie) is a axon fragment?

Phenotype 2: Myelin-Remak hybrids with long, thin processes connecting them.
On the other hand, the phenotype of Schwann cells making long, thin processes that span multiple sheaths (e.g. one myelin sheath and one Remak bundle) is very interesting and the authors' quantification of this is rigorous. The fact that this phenotype is seen even in the absence of mTor

does strongly suggest a novel role of Fbxw7 in Schwann cell morphology. However, how can we be sure that the same SC is making both a myelin sheath and a Remak bundle- couldn't this also be due to an aberrant process being extended between neighboring myelinating SC and Remak SC (in this case the two different types of myelin come from different cells, like normal, but there is some abnormal membrane protrusion that can appear to connect them). Again, this might also be more clearly resolved by sparse labeling of Schwann cells in vivo. It is also possible that this will be clearer to see if the authors present paired raw TEM micrographs in addition to the false-colored images.

Minor points:

1. The authors describe an oligodendrocyte-like behavior for Fbxw7 mutant SCs in myelination of multiple axons, but do not comment on the role of this gene in oligodendrocyte myelination capacity. Can the authors comment on if mature oligodendrocytes express Fbxw7 during myelination and the global or OL specific knockouts of Fbxw7 change the number of myelin sheaths formed by OLs.

2. From the images in Figure 1.A-C, it seems that there are more myelinated axons in the controls in comparison to heterozygous or null Fbxw7 mutants. This is in contrast with the data displayed on Figure1E. Are these representative images?

3. Can the authors comment on the overall health and behavior of these mutants? It will be interesting to know if the SC specific deletion of Fbxw7 leads to any peripheral sensory phenotypes.

4. Introduction. "promyelinating SCs are associated 1:1 with large-caliber axons (<1 μm in diameter)" should read ">1 μm in diameter".

Citations
1) Sander et al. (2000) Clinical syndromes associated with tomacula or myelin swellings in sural nerve biopsies. J Neurol Neurosurg Psychiatry. 68: 483-8.
2) Domènech-Estévez et al. (2016). Akt Regulates Axon Wrapping and Myelin Sheath Thickness in the PNS. J Neurosci. 36: 4506-21.

We thank the reviewers for their helpful critiques and suggestions, which have significantly improved our manuscript. We have performed several more experiments and added substantial new data to the revised manuscript, which have strengthened our conclusions and enhanced the manuscript. Below, please find our point-by-point responses to the issues raised by the reviewers. We also note that in the revised version, all major text changes in the main body of the manuscript are marked in blue.

Your manuscript entitled "Fbxw7 is a critical regulator of Schwann cell myelinating potential" has now been seen by 3 referees, whose comments are appended below. You will see from their comments copied below that while they find your work of considerable potential interest, they have raised quite substantial concerns that must be addressed. In light of these comments, we cannot accept the manuscript for publication, but would be interested in considering a revised version that addresses these serious concerns.

We hope you will find the referees' comments useful as you decide how to proceed. Specifically, we require that you add the sparse labeling experiment as suggested by reviewer #3 to convince the reviewers of the multiple-axon myelination phenotype. We also ask that you add western blotting controls per concerns of reviewer #2. Finally, we ask you to better explain the significance of the study in your point-by-point and by editing the manuscript, as suggested by reviewer #2, and to help convince reviewer #1 of the significance. We'd additionally encourage you to expand the molecular mechanism, if possible.
Response: We have addressed all of these main concerns, as detailed below.

Reviewers' comments:

Reviewer #1 (Remarks to the Author):

I have seen a prior version of this paper before for another journal and maintain that this is a very well presented study. It reports a hypermyelination phenotype of the PNS in zebrafish caused by a mutation in the gene for FBXW7, which is then more systematically studied in mice with the conditional gene targeting of Schwann cells.
Response: We note that zebrafish data were not/are not included in this manuscript, which instead only includes mouse models.

FBXW7 is member of the E3 ubiquitin ligase complex and known to have mTOR as a downstream target. The CNS phenotype of these mutants was previously published. Here, the paper concentrates on the description of elevated mTOR signaling in Schwann cells, thereby confirming similar observations in mouse mutants with loss of Pten or increased Akt expression. The authors also report a few novel features, such as multi-axonal myelination profiles. However, the latter appears not be caused by mTOR activation, as heterozygosity of mTOR in conditional Fbxw7 double mutants does not interfere with these phenotypes. What other targets of FBXW7 are responsible remains unknown.

I also repeat one critical comment that while the paper is nicely written and provides very beautiful data, the question comes up what is really new and could not be predicted from the known facts that (a) FBXW7 is required to degrade mTOR and (b) abnormally high mTOR signaling causes the hypermyelination phenotype detailed in this and other manuscripts.
Response: The reviewer is correct that Fbxw7 has already been shown to degrade mTOR and that high levels of mTOR (such as those observed in our *Fbxw7* mutants) is known to cause hypermyelination. However, the main novel finding of the study is that loss of Fbxw7 in Schwann cells confers the remarkable ability to myelinate multiple axons *in vivo* and *in vitro*. Moreover, loss of Fbxw7 leads to a never-before-reported non-myelinating Schwann cell phenotype, in which a single Schwann cell displays characteristics of both a myelinating Schwann cell and a non-myelinating Schwann cell. Importantly, our epistasis experiments indicate that neither of these phenotypes is regulated by mTOR.

To reiterate: the multi-axon myelinating phenotype and the myelinating/non-myelinating Schwann cell "hybrid" are both "really new and could not have been predicted from the known facts." We show that loss of a single gene fundamentally changes cell biological properties of Schwann cells previously thought to be immutable.

I am still missing some analysis of FBXW7 itself as a Schwann cell protein and its interaction with relevant target proteins. When and where is it expressed? Is the FBXW7 function itself regulated, e.g. in Schwann cell development, or is the protein constitutively active in the lineage?
Response: We tested all available commercial antibodies to Fbxw7 and unfortunately, did not find any that were specific for IHC. To circumvent this, we now include RNAseq data (revised Figure 6) that demonstrates *Fbxw7* mRNA levels are constant in early (P3) and more mature (P21) peripheral nerve.

What are the (other) targets of FBXW7-dependent ubiquitination in Schwann cells?
Response: Revised Figure 6 also highlights several targets of Fbxw7 that are present in developing nerve, one of which, c-Jun, is upregulated at the protein level in *Fbxw7* knock out Schwann cells. We have also expanded the text (p. 11) to indicate that as many as 1,700 Fbxw7 targets exist in the human genome; thus, an exhaustive analysis of all potential targets is beyond the scope of this manuscript.

Several other minor comments that I had previously made are all taken care of in this extensively revised manuscript.

Reviewer #2 (Remarks to the Author):

The manuscript by Harty et al., attempts to demonstrate a novel role for Fbxw7 as a important regulator of Schwann cell myelinating potential. Deletion of Fbxw7 results in the myelination of multiple axons and a hypermyelination phenotype. The authors demonstrate that mTOR dysregulation underlies the hypermyelination phenotype, however the ability of SCs to myelinate multiple axons was independent of mTOR activation. This is a fascinating study and one that

speaks to the intrinsic differences between oligodendrocytes and SCs. The experiments are well detailed and rigorous. However, there are just a few suggestions that should be addressed to help make the conclusions even more compelling.

1. The authors propose that Fbxw7 is an upstream suppressor of mTOR expression/activation and that this is likely the result of Akt. Can the authors provide p-Akt levels from the sciatic nerve lysates? Additionally, the Western blot of mTOR is a bit saturated and the only observable differences are based on the decrease in the tub loading control. It would benefit the rigor of the study to show multiple lanes of sciatic nerve samples and maybe include additional control proteins like various myelin proteins, Krox20 and maybe even Oct6.
Response: We now show multiple lanes of sciatic nerve samples (revised Figure 4) as requested by the reviewer. Krox20 protein is examined by IHC *in vitro* as is now included in revised Figure 3.

2. The title of the manuscript does not fully convey the data presented nor the novelty of the study. Fbxw7 acts as a suppressor of mTOR expression activation and mediates proper myelin deposition/thickness. Additionally, it ensures that SCs myelinate a single axon. As it stands, the title does not describe the surprising findings in the manuscript.
Response: We appreciate this suggestion and have changed the title from *Fbxw7 is a critical regulator of Schwann cell myelinating potential* to *Schwann cells myelinate multiple axons in the absence of Fbxw7*.

3. As axonal signals are especially important for the induction of myelination, what axonal signals contribute to Fbxw7 activation/expression to achieve the proper g-ratios?
Response: We now clarify that *Fbxw7* mutant Schwann cells do not myelinate axons $\leq$ 1 µm any more frequently than control Schwann cells. This indicates that Neuregulin/Erbb signals are intact in the mutants (p. 5). Myelin thickness (aberrant g-ratios) are impacted via dysregulated mTOR activity, as described in the text and in several figures (Figures 4, 5, S4).

If the Fbxw7 SCs resemble SCs after injury, what extrinsic cues contribute to this conversion? Additional discussion on these topics would be helpful.
Response: The extrinsic signal(s) that converts a resting Schwann cell to a repair Schwann cell is completely unknown. Although solving this important mystery is outside of the scope of our present manuscript, we did begin to dissect what might be intrinsic to the Schwann cell that contributes to a repair Schwann cell-like phenotype. The transcription factor c-Jun is required for the conversion of resting Schwann cells to repair Schwann cells, and c-Jun(+) repair Schwann cells assume a branched morphology reminiscent of *Fbxw7* mutant Schwann cells (Jessen & Mirsky labs). We now show that *Fbxw7* mutants Schwann cells have increased levels of c-Jun compared to control Schwann cells. This result both deepens the mechanism reported in our manuscript and sheds light on the unique cellular phenotypes we observe in *Fbxw7* mutant Schwann cells.

Reviewer #3 (Remarks to the Author):

In their manuscript “Fbxw7 is a critical regulator of Schwann cell myelinating potential” the authors describe a novel role for the gene Fbxw7 in regulating myelination by Schwann cells (SCs). Fbw7 mutants display an increased number of SCs, a higher proportion of myelinated axons during early development and thicker myelin sheaths. The authors show that these hypermyelination phenotypes are due to the dysregulation of mTOR pathway. Intriguingly, Fbw7 mutants exhibit additional mTOR-independent phenotypes that the authors interpret as SCs myelinating multiple axons or forming both a myelin sheath and a Remak bundle, with abnormal cytoplasmic processes connecting these different sheaths. If true it would suggest that loss of Fbxw7 causes SCs to adopt an oligodendrocyte-like morphology and function, and completely novel phenotype. However, I have some concerns about the interpretation of the phenotype and think that additional work would strengthen this very interesting manuscript.

Major points:
Phenotype 1: Multiple axons myelinated.
I am not entirely convinced that Fbxw7 causes SCs to myelinate multiple axons. In the serial EM sections presented as Supplemental Movie 1, the authors label a SC that they interpret as myelinating two axons (starting at first frame). The labelled SC seems to contain two axons from the first frame, but, the deeper sections reveal that the bottom right myelin sheath closes in on itself and then disappears. If this was an axon myelinated by the SC, the deeper sections would show unmyelinated segments of that axon. Three-dimensionally, my interpretation of this sheath that closes in on itself as you section deeper is that this was a “dome” or “sphere” of myelin and not a normal cylindrical myelin sheath around an intact axon. This phenotype seems likely to be a result of extreme myelin outfolding/infolding, similar to the range of phenotypes described before for other regulators of the PTEN/Akt/mTor pathways and clinically in Charcot Marie Tooth disease and various peripheral neuropathies. See for example Sander et al. and Demonech-Estevez et al. (citations below).

Indeed, following other Schwann cells through the movie, we see additional examples of complex myelin outfoldings that seem to appear and disappear as we traverse the length of the nerve: e.g. the SC on the bottom left that gets highlighted with yellow arrows has another large “myelin dome”, visible from beginning of movie to ~4s when it connects to the main sheath; it also has a “myelin sphere” visible from 9s to 12s.
Response: The original video is no longer included in our study. A new video (Movie 1) instead shows non-myelinating and myelinating Schwann cell cytoplasmic linkage.

This is certainly an interesting phenotype, but I do not think it is fair to conclude that Fbxw7 Schwann cells make multiple myelin sheaths. All of the multiple myelin sheaths seen in single section TEM micrographs in the figures/quantification could just as easily be due to catching a complex outfolding in a section where it appears to be multiple axons. Perhaps the clearest and easiest way to see if these mutant Schwann cells really do myelinate multiple axons and the extent to which this happens would be to sparse label them (genetically, virally, or possibly in a teased nerve preparation) and visualize using light microscopy.
Response: We agree with the reviewer that it is critical to demonstrate the novel multi-axon myelination phenotype observed in *Fbxw7* mutant Schwann cells by an additional method

besides electron microscopy. To address this, we approached the lab of Jonah Chan (UCSF) to test if the multiaxon myelination phenotype could be recapitulated: a) *in vitro*; b) by an independent laboratory at a separate institution. The Chan lab also observed striking multi-axon myelinating by *Fbxw7* mutant Schwann cells in myelinating DRG co-cultures. These results are included in the revised Figure 3.

Also, is there any evidence of axon loss in these mutants? Is it possible that myelin around a structure that appears axonal but does not extend cylindrically (as in the movie) is a axon fragment?
Response: We did not observe axon loss in Schwann cell conditional *Fbxw7* mutants. These data are now included in Figure S1.

Phenotype 2: Myelin-Remak hybrids with long, thin processes connecting them.
On the other hand, the phenotype of Schwann cells making long, thin processes that span multiple sheaths (e.g. one myelin sheath and one Remak bundle) is very interesting and the authors' quantification of this is rigorous. The fact that this phenotype is seen even in the absence of mTor does strongly suggest a novel role of Fbxw7 in Schwann cell morphology. However, how can we be sure that the same SC is making both a myelin sheath and a Remak bundle- couldn't this also be due to an aberrant process being extended between neighboring myelinating SC and Remak SC (in this case the two different types of myelin come from different cells, like normal, but there is some abnormal membrane protrusion that can appear to connect them). Again, this might also be more clearly resolved by sparse labeling of Schwann cells in vivo. It is also possible that this will be clearer to see if the authors present paired raw TEM micrographs in addition to the false-colored images.
Response: We now pair raw TEM along with false-colored images (Figure 2H-I'; Figure 3F'; Movie S1).

Minor points:

1. The authors describe an oligodendrocyte-like behavior for Fbxw7 mutant SCs in myelination of multiple axons, but do not comment on the role of this gene in oligodendrocyte myelination capacity. Can the authors comment on if mature oligodendrocytes express Fbxw7 during myelination and the global or OL specific knockouts of Fbxw7 change the number of myelin sheaths formed by OLs.
Response: In the present manuscript, we cite previous studies from our lab (Sanchez et al., 2017) and the Appel lab (Snyder et al. 2012; Kearns et al., 2015) showing hypermyelination in global *fbxw7* zebrafish mutants. Although we continue to dissect the interesting function of Fbxw7 in oligodendrocyte biology and CNS myelination, these studies are outside of the scope of the present manuscript.

2. From the images in Figure 1.A-C, it seems that there are more myelinated axons in the controls in comparison to heterozygous or null Fbxw7 mutants. This is in contrast with the data displayed on Figure1E. Are these representative images?
Response: Panels in revised Figure 1A-C now show more representative images.

3. Can the authors comment on the overall health and behavior of these mutants? It will be interesting to know if the SC specific deletion of Fbxw7 leads to any peripheral sensory phenotypes.
Response: We note in the revised manuscript that conditional mutants are viable and fertile with no gross phenotypes (p. 4). We also performed a battery of motor and sensory behavioral analyses at six months of age and found some modest behavioral defects. These data are included in the revised manuscript (p. 7-8; Figure S3).

4. Introduction. “promyelinating SCs are associated 1:1 with large-caliber axons (<1 μm in diameter)” should read “>1 μm in diameter”.
Response: We have corrected this error.

Citations

1) Sander et al. (2000) Clinical syndromes associated with tomacula or myelin swellings in sural nerve biopsies. J Neurol Neurosurg Psychiatry. 68: 483-8.
2) Domènech-Estévez et al. (2016). Akt Regulates Axon Wrapping and Myelin Sheath Thickness in the PNS. J Neurosci. 36: 4506-21.

REVIEWERS' COMMENTS:

Reviewer #1 (Remarks to the Author):

The authors have adequately addressed the questions that I had.

Reviewer #3 (Remarks to the Author):

My previous major concern was that the author's conclusion that Fbxw7-deficient Schwann cells myelinate multiple axons was not definitively demonstrated, and their data left room for ambiguity. In the revised manuscript this concern has been addressed with new data, most importantly myelinating cocultures with Fbxw7-deficient Schwann cells, a system where it is straightforward to resolve between different Schwann cells. Strikingly, Fbxw7-deficient Schwann cells adopt a multi-process morphology on DRG axons that indeed makes them resemble oligodendrocytes. I think this work is highly novel, rigorous, and that the authors have addressed most of the major critiques of I and the other reviewers. I also like the proposed link between Fbxw7 deficiency and the recently described multipolar "repair" Schwann cell, and the molecular link between them (elevated c-Jun) is a compelling addition to the paper. The authors also added rigorous behavioral/functional quantification of mice, showing defects in several metrics due to loss of Fbxw7. These further strengthen the paper.

My only remaining concern is really one of semantics/interpretation. While I completely believe the vast and orthogonal data presented that Fbxw7-deficient Schwann cells can interact with and ensheath multiple axons, I still hold that the data do not unambiguously show that these mutant Schwann cells are able to MYELINATE multiple axons, where "myelinate" refers to making multilamellar compact myelin. The new coculture data shows compelling MBP staining that is consistent with this hypothesis, but actual myelin is not shown here and MBP staining in culture does not necessarily reflect compact myelin at the ultrastructural level. In addition, the example micrographs where it might look like two axons are myelinated by the same Schwann cell (Fig. 2B,C,I; Fig. 5B) still suffer from the same ambiguity as the previous version of this manuscript: what is the evidence that these are myelin sheaths around axons? The FIB-SEM supplemental movie in the previous submission contained frames that looked very similar to these micrographs, with what appeared to be two myelin sheaths contained within the same Schwann cell's membrane and basal lamina. However, going above or below these frames revealed that what appeared to be a "tube" of myelin often was a complex outfolding that closed off on itself above or below, so there was no intact axon traversing the myelin- this cannot be called myelination if there is no axon inside. Simply omitting the previous movie in this resubmission is not compelling evidence that compact myelin is ever formed around multiple axons. (The new movie shows a nice example of the other, completely convincing phenotype: that these mutant Schwann cells can ensheath one axon while myelinating another). My recommendation is to soften the language, for example claiming in the title that "Schwann cells ensheath multiple axons in the absence of Fbxw7" and interpreting the data in 2B,C,I, etc. that these Schwann cells "appear to" form myelin around multiple axons.

To be clear, I don't think that this semantic argument weakens the novelty or broad appeal of this paper, which is even more interesting with the new data. But extraordinary claims require extraordinary proof, and fully unambiguous data showing "myelination" of multiple axons are still not provided.

Other minor points:
Fig 3d: what is the Y axis normalized to? E.g. # of multipolar Schwann cells per some area?
Fig. 6A: Labeling of the bar graph could be improved for clarity, e.g. by having the paired P3 and P21 bars of each individual gene touching and gene names centered under each pair of bars.

We thank the reviewers for their continued time spent on our manuscript. Below, please find our response to Reviewer #3.

REVIEWERS' COMMENTS:

Reviewer #1 (Remarks to the Author):

The authors have adequately addressed the questions that I had.

Reviewer #3 (Remarks to the Author):

My previous major concern was that the author's conclusion that Fbxw7-deficient Schwann cells myelinate multiple axons was not definitively demonstrated, and their data left room for ambiguity. In the revised manuscript this concern has been addressed with new data, most importantly myelinating cocultures with Fbxw7-deficient Schwann cells, a system where it is straightforward to resolve between different Schwann cells. Strikingly, Fbxw7-deficient Schwann cells adopt a multi-process morphology on DRG axons that indeed makes them resemble oligodendrocytes. I think this work is highly novel, rigorous, and that the authors have addressed most of the major critiques of I and the other reviewers. I also like the proposed link between Fbxw7 deficiency and the recently described multipolar "repair" Schwann cell, and the molecular link between them (elevated c-Jun) is a compelling addition to the paper. The authors also added rigorous behavioral/functional quantification of mice, showing defects in several metrics due to loss of Fbxw7. These further strengthen the paper.

My only remaining concern is really one of semantics/interpretation. While I completely believe the vast and orthogonal data presented that Fbxw7-deficient Schwann cells can interact with and ensheath multiple axons, I still hold that the data do not unambiguously show that these mutant Schwann cells are able to MYELINATE multiple axons, where "myelinate" refers to making multilamellar compact myelin. The new coculture data shows compelling MBP staining that is consistent with this hypothesis, but actual myelin is not shown here and MBP staining in culture does not necessarily reflect compact myelin at the ultrastructural level. In addition, the example micrographs where it might look like two axons are myelinated by the same Schwann cell (Fig. 2B,C,I; Fig. 5B) still suffer from the same ambiguity as the previous version of this manuscript: what is the evidence that these are myelin sheaths around axons? The FIB-SEM supplemental movie in the previous submission contained frames that looked very similar to these micrographs, with what appeared to be two myelin sheaths contained within the same Schwann cell's membrane and basal lamina. However, going above or below these frames revealed that what appeared to be a "tube" of myelin often was a complex outfolding that closed off on itself above or below, so there was no intact axon traversing the myelin- this cannot be called myelination if there is no axon inside. Simply omitting the previous movie in this resubmission is not compelling evidence that compact myelin is ever formed around multiple axons. (The new movie shows a nice example of the other, completely convincing phenotype: that these mutant Schwann cells can ensheath one axon while myelinating another). My recommendation is to soften the language, for example claiming in the title that "Schwann cells ensheath multiple axons in the absence of Fbxw7" and interpreting the data in 2B,C,I, etc. that these Schwann cells "appear to" form myelin around multiple axons.
We have edited the language throughout the manuscript as suggested by the reviewer and the editor.

To be clear, I don't think that this semantic argument weakens the novelty or broad appeal of this paper, which is even more interesting with the new data. But extraordinary claims require

extraordinary proof, and fully unambiguous data showing “myelination” of multiple axons are still not provided.

Other minor points:
Fig 3d: what is the Y axis normalized to? E.g. # of multipolar Schwann cells per some area?
We have clarified this in the figure legend.

Fig. 6A: Labeling of the bar graph could be improved for clarity, e.g. by having the paired P3 and P21 bars of each individual gene touching and gene names centered under each pair of bars.
We have modified this figure as suggested.
